# Characterization of *Leishmania donovani* Aquaporins Shows Presence of Subcellular Aquaporins Similar to Tonoplast Intrinsic Proteins of Plants

**DOI:** 10.1371/journal.pone.0024820

**Published:** 2011-09-28

**Authors:** Neha Biyani, Swati Mandal, Chandan Seth, Malika Saint, Krishnamurthy Natarajan, Indira Ghosh, Rentala Madhubala

**Affiliations:** 1 School of Life Sciences, Jawaharlal Nehru University, New Delhi, India; 2 Laboratory of Eukaryotic Gene Regulation, School of Life Sciences, Jawaharlal Nehru University, New Delhi, India; 3 School of Computational and Integrative Sciences, Jawaharlal Nehru University, New Delhi, India; Technion-Israel Institute of Technology Haifa 32000 Israel., Israel

## Abstract

*Leishmania donovani*, a protozoan parasite, resides in the macrophages of the mammalian host. The aquaporin family of proteins form important components of the parasite-host interface. The parasite-host interface could be a potential target for chemotherapy. Analysis of *L. major* and *L. infantum* genomes showed the presence of five aquaporins (AQPs) annotated as AQP9 (230aa), AQP putative (294aa), AQP-like protein (279aa), AQP1 (314aa) and AQP-like protein (596aa). We report here the structural modeling, localization and functional characterization of the AQPs from *L. donovani*. LdAQP1, LdAQP9, LdAQP2860 and LdAQP2870 have the canonical NPA-NPA motifs, whereas LdAQP putative has a non-canonical NPM-NPA motif. In the carboxyl terminal to the second NPA box of all AQPs except AQP1, a valine/alanine residue was found instead of the arginine. In that respect these four AQPs are similar to tonoplast intrinsic proteins in plants, which are localized to intracellular organelles. Confocal microscopy of *L. donovani* expressing GFP-tagged AQPs showed an intracellular localization of LdAQP9 and LdAQP2870. Real-time PCR assays showed expression of all aquaporins except LdAQP2860, whose level was undetectable. Three-dimensional homology modeling of the AQPs showed that LdAQP1 structure bears greater topological similarity to the aquaglyceroporin than to aquaporin of *E. coli*. The pore of LdAQP1 was very different from the rest in shape and size. The cavity of LdAQP2860 was highly irregular and undefined in geometry. For functional characterization, four AQP proteins were heterologously expressed in yeast. In the *fps1Δ* yeast cells, which lacked the key aquaglyceroporin, LdAQP1 alone displayed an osmosensitive phenotype indicating glycerol transport activity. However, expression of LdAQP1 and LdAQP putative in a yeast *gpd1Δ* strain, deleted for glycerol production, conferred osmosensitive phenotype indicating water transport activity or aquaporin function. Our analysis for the first time shows the presence of subcellular aquaporins and provides structural and functional characterization of aquaporins in *Leishmania donovani*.

## Introduction

The protozoan parasite *Leishmania* is the causative agent of kala-azar and is responsible for a variety of clinical manifestations. Visceral leishmaniasis (VL) is caused by *L. donovani* in the Indian sub-continent. Pentavalent antimonials (SbV) are the first line of drug used in the treatment against all forms of leishmanial infections. Resistance to this drug is becoming a major barrier in the treatment of VL in many endemic regions particularly in India [1]. The parasite life cycle consists of two morphologically distinct stages. The promastigote forms live inside the gut of the sandfly and the amastigote forms reside in the macrophages of the mammalian host. Parasite-host interface could be a potential target for chemotherapy [2].

The aquaporin family of proteins form important components of the parasite-host interface [3]. These channels are widely distributed in all kingdoms of life, including bacteria, plants, and mammals [4]. Aquaporins (AQPs) are a family of membrane channels primarily responsible for conducting water across cellular membranes (orthodox aquaporins) or which pass preferably uncharged polar solutes like glycerol and urea (aquaglyceroporins) [5].

Various genes encoding aquaporin channels have been identified in the protozoan genomes and the potential of these protozoan channels for use as a target or entry pathway for chemotherapeutic compounds is under investigation. The *Leishmania major* genome encodes for five AQPs: LmAQP1, LmAQPα, LmAQPβ, LmAQPγ and LmAQPδ [6]. Only LmAQP1 has been studied in some detail, while the role of the other LmAQPs is yet to be established. LmAQP1 belongs to the intermediate class of water channels; its water conduction capacity is 65% that of AQP1, which is a classical water channel [7]. LmAQP1 also conducts glycerol, glyceraldehyde and dihydroxyacetone. In contrast, there is negligible urea conduction by LmAQP1, and this property probably helps the parasite to survive the hostile environment of liver cells. It also plays an important physiological role in water and solute transport, volume regulation and osmotaxis [8].

In the yeast *S. cerevisiae*, two aquaglyceroporin genes *FPS1* and *YFL054C* have been identified [9], [10]. However, a functional role in active glycerol transport was shown only for *FPS1* [9] but no physiological role has been ascribed to *YFL054C* [11]. When the yeast strains are cultured in the presence of high concentration of osmolytes such as sorbitol, the strains accumulate intracellular glycerol in response to the extracellular stress. However, when cells are returned to medium with no osmolytes, the intracellular glycerol is released via the Fps1p channel [9]. In the absence of the Fps1p channel, however, cells are unable to release the accumulated glycerol and consequently the cells show poor growth. This system has been exploited by several groups to study aquaglyceroporin genes from *T. brucei* [12], cauliflower [13], Arabidopsis [14] and human [15]. The yeast *GPD1* gene codes for glycerol-3-phosphate dehydrogenase that is involved in glycerol production [16], deletion of which confers osmosensitivity on yeast strains [15]. On exposure to hyperosmotic stress, heterologous genes when expressed in a *gpd1Δ* strain display slow growth phenotype due to water loss [15], thus uncovering the water transport activity or aquaporin function.

LmAQP1 has been shown to be a metalloid transporter [17], [18]. Our studies with large number of antimony -resistant clinical isolates indicated that while down regulation of *AQP1* may be one of the mechanisms of antimony resistance it is however not an invariable feature of such resistance [18], [19]. Moreover, the *Leishmania* parasite never encounters metalloids during its life cycle, therefore besides metalloid transport AQPs may also serve a physiological function in water homeostasis, glycerol transport, volume regulation and osmotaxis [8].

In the present study we report the identification and characterization of five aquaporin genes from *L. donovani* (*LdAQP1, LdAQP9, LdAQP2860, LdAQP2870 and LdAQP putative*). Localization studies show sub-cellular localization of two of the *Leishmania* AQPs. We for the first time report that *L. donovani* has subcellular aquaporins with deviated second NPA box similar to tonoplast intrinsic proteins of plant. Functional characterization of AQP genes was done by heterologous expression in *Saccharomyces cerevisiae*. We have also built three dimensional structures using homology modeling tools and analyzed the channel or pore characteristics with regard to their specific chemical portability.

## Results

### Isolation of *L. donovani* aquaporin (LdAQP) genes, sequence analysis and structural modeling

Five aquaporin sequences have been identified in each of the *L. infantum* and *L. major* genomes (www.ebi.ac.uk/parasites/LGN/). BLAST analysis of *L. major* and *L. infantum* genomes revealed that the AQP genes are present on chromosomes number 22 (*AQP putative*: LmjF.22.1420, LinJ.22.1270), 32 (*AQP-like protein*: LmjF.32.2370, LinJ.32.2500), 32 (*AQP-like protein*: LmjF.32.2380, LinJ.32.2510), 34 (AQP9: LmjF.34.3850, LinJ.34.3660) and 31 (*AQP1*: LmjF.31.0020, LinJ.31.0030). We have cloned four AQP genes from *L. donovani*. Sequence analysis, database search, and alignment of the *L. donovani* AQPs amino acid sequence were performed as described in the materials and methods. The sequences have been submitted to NCBI data base as *AQP9* (Gen Bank Accession ID: GU199598.1), *AQP putative* (Gen Bank Accession ID: GU199596.1), *AQP2870* (Gen Bank Accession ID: GU199597.1), *AQP1* (Gen Bank Accession ID: EF600686.1). In the present study the sequence obtained for *AQP2870* of *L. donovani* corresponded to *AQP-like protein* LmjF.32.2380 and LinJ.32.2510. *LdAQP2860* sequence shown in the present study for alignment and structural analysis was that of *L. infantum AQP-like protein*, LinJ.32.2500 (The Gen Bank Accession ID: XM_001467890.1). The open reading frames code for proteins of AQP9 (230aa), AQP putative (294aa), AQP2870 (279aa), AQP1 (314aa) and AQP2860 (596aa).

**Figure 1:**
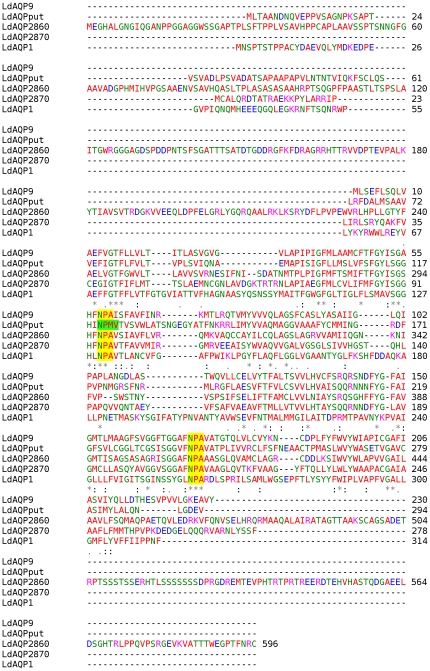
Multiple sequence alignment of complete amino acid sequence of *L. donovani* AQPs using ClustalW. The small (small+hydrophobic (incl.aromatic -Y)) have been marked in RED, acidic residues in BLUE, basic residues in MAGENTA, GREEN marks hydroxyl+amine+basic - Q and others in Gray. “*” means that the residues or nucleotides in that column are identical in all sequences in the alignment. “:” means that conserved substitutions have been observed according to the COLOUR table above. “.” means that semi-conserved substitutions have been observed. NPA motif has been highlighted with yellow, however, non canonical motifs in filter have been marked in green. The NCBI accession numbers of the protein sequences are same as mentioned in Figure S1.

A very low degree of amino acid sequence similarity is observed in *L. donovani* aquaporins due to the variation in the length of the protein sequences. Hence multiple sequence alignments done by BLOSUM62 matrix did not yield high sequence similarity values. However, the nature of amino acids (hydrophobicity, hydrophilicity, polar, non polar character) required for forming the typical hour-glass structure, appear to be conserved. Figure S1 shows the multiple sequence alignment using CLUSTALW of all five *L. donovani* AQPs, all five *L. major* AQPs and other AQPs with known crystal structures (Table S1). *L. donovani* and *L. major* AQPs were found to group alike. Figure 1 shows the multiple sequence alignment using CLUSTALW of all *L. donovani* AQPs only. The sequences were similar in the trans-membrane helical region and aligned at the NPA (asparagine-proline-alanine) motifs. Most aquaporins have two highly conserved hydrophobic NPA boxes that form a pore for uptake of water and/or glycerol and urea. LdAQP1, LdAQP9, LdAQP2860 and LdAQP2870 were found to have the canonical NPA-NPA motif in the filter. LdAQP putative has a non canonical NPM-NPA motif (Table 1). The upstream of the first NPA box in the four LdAQPs (AQP9, AQP putative, AQP2870 and AQP1) is particularly conserved: SG(A/G)HXNPA (Table 2). The downstream sequence of the second NPA box is reported to be highly conserved in most AQPs (NPAR(D/S/A). In case of LdAQP1 the hydrophobic groups downstream of the second NPA box have charged amino acids i.e. NPARD. The arginine (R) is particularly important as it serves as the selective filter in AQP and may make it a glyceroporin (Table 1). AQP-like sequences with deviated NPA boxes have been reported in the plant aquaporins [20], [21]. The second NPA motif having either NPAVA or NPAAA or NPAIA has been reported in the plant SIPs (small basic intrinsic proteins) or TIPs (tonoplast integral proteins). These plant aquaporins are subcellular and are known to have water channel function. Replacement of arginine (R) with valine (V) or alanine (A) could alter the pore structure. Four of the identified LdAQPs, with the exception of LdAQP1 have second NPA box similar to the plant TIPs (Table 2). Table 2 shows upstream and down- stream sequences of the two NPA boxes of LdAQPs.

**Table 1:** The transmembrane helical region ranges and the position of the selectivity filter predicted after detailed analysis of *L. donovani* AQP protein sequences.

*L. donovani* AQP (Length)	TM1	TM2	TM3	NPA1	NPA2	TM4	TM5	TM6
AQP1 (314aa)	63–82 (20)	104–123 (20)	145–166 (22)	130–132 NPA	260–262 NPA	206–223 (18)	241–258 (18)	288–307 (20)
AQP9 (230aa)	9–28 (20)	42–63 (22)	77–96 (20)	58–60 NPA	170–172 NPA	112–133 (22)	150–169 (20)	194–213 (20)
AQP- putative (294aa)	68–90 (23)	99–123 (25)	144–165 (22)	120–122 NPM	239–241 NPA	183–202 (20)	213–232 (20)	267–286 (20)
AQP2860 (596aa)	237–354 (18)	281–303 (22)	313–335 (20)	297–299 NPA	408–410 NPA	349–372 (20)	382–402 (20)	432–451 (20)
AQP2870 (279aa)	32–52 (21)	68–92 (24)	111–132 (22)	95–97 NPA	210–210 NPA	153–172 (20)	193–212 (20)	232–253 (22)

Length of the transmembrane helix (TM1–TM6) is mentioned in parenthesis. AQP putative appears to have NPM instead of NPA motif as indicated.

**Table 2:** Sequence alignment of *L. donovani* aquaporins at the first and second NPA box.

	First NPA box	Second NPA box
AQP9	FGYISGAHF**NPA**ISFAVFINR	MAAGFSVGGFTGGAF**NPA**VATGTQLVL**C**
AQP Put.	LVFSFGYLSGGHI**NPMV**TVSVWLAT	LCGGLTCGSISGGVF**NPA**VATPLIVVRC
AQP2860	SMIFTFGYISGSHF**NPA**VSIAVFLVR	SAGSASAGRISGGAF**NPA**AASGLQVAM**C**
AQP2870	VLIFMFGYISGGHF**NPA**VTFAVVMIR	LASQYAVGGVSGGAF**NPA**VAAGLQVTKF
AQP1	IGLFLSMAVSGGHL**NPA**VTLANCVFG	FVIGITSGINSSYGL**NPA**RDLSPRILSA
TIP1.1	GANISGGHV**NPA**VTFGAFIG	GGAFSGASM**NPA**VAFGPAVVSW
SIP1.1	TVIFGSASF**NPT**GSAAFYVA	GSKYTGPAM**NPA**IAFGWAYMYS

The sequences around the two NPA (underlined) are aligned. NPAs (asparagines-proline-alanine) are underlined. The second NPA box of *L. donovani* AQPs with the exception of AQP1 are similar to tonoplast integral protein (TIP1.1) which is localized to intracellular organelles. Small basic intrinsic proteins (SIP1.1) are plant subcellular aquaporins. Tip1.1 sequence is from a plant *Arabidopsis thaliana* (Q9ZV07). SIP1.1 sequence is also from *Arabidopsis thaliana* (Q9M8W5). The downstream sequence of the second NPA box of AQP1 has arginine (R) and is replaced by valine (V) or alanine (A) in the remaining LdAQPs.

**Figure 2:**
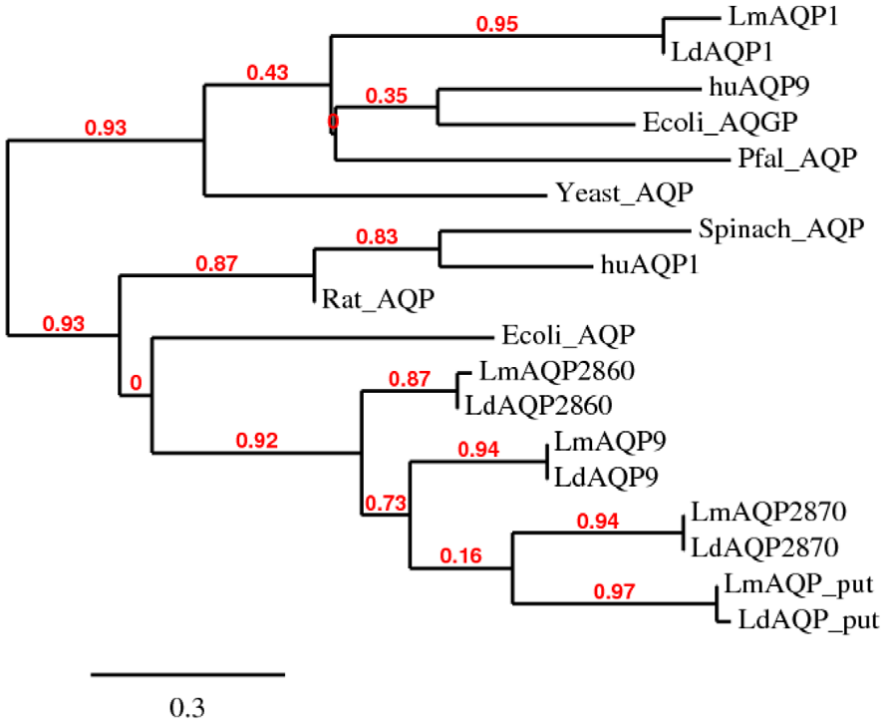
Phylogram derived from the multiple sequence alignment for the complete amino acid sequence of AQPs using ClustalW. Branch support values are shown in red. [Branch support is quantified as the extra length needed to lose a branch in the consensus of near-most-parsimonious trees. This approach is based solely on the original data, as opposed to the data perturbation used in the bootstrap procedure].

A brief phylogenetic analysis was carried out to predict the position of the *L. donovani* AQPs in the evolutionary tree and to find its relatedness to the AQPs with known crystal structures. The phylogram branched in to two major clusters. One cluster consisted of LdAQP1 and its homologues, while the other contained all the other *L. donovani* AQPs i.e. LdAQP9, LdAQP putative (LdAQP put), LdAQP2860 and LdAQP2870 (Fig. 2). In the first cluster, LdAQP1 was found to be in close relation with human AQP9. Since the crystal structure of the human AQP9 is not known, we therefore used the crystal structure of *E. coli* AQGP (PDB ID: 1LDA), the close homolog for comparison. LdAQP1 was also found to be close to the *P. falciparum* AQP and the yeast AQP. The leaves for LdAQP9, LdAQP putative, LdAQP2860 and LdAQP2870 in the second cluster were found to be close to each other. The nearest leaf for the known crystal structure to these was of *E. coli* AQP (2ABM). Close to this branch is the branch containing rat AQP, human AQP1, and spinach AQP. Yeast AQP is close to cluster 1 (containing LdAQP1) and cluster 2. The related known structures as observed by phylogram were used as structural templates for model building of *L. donovani* family of AQPs (Table S1).

Hydropathy indexing for *L. donovani* AQP amino acid sequences was done using ProtScale (databank based method) [22] , TMHMM server (HMM method) [23] and OCTOPUS (neural network method) [24]. These were chosen so as to cover the diverse range of algorithms from database type to artificial network based prediction type (Fig. S2.1 and Fig. S2.2). Each of this methods predicted six major transmembrane helices and two additional minor helices. The consistent results signify the accuracy of prediction. The OCTOPUS analysis was significant since it showed the presence of two relatively small peaks. One of them was present in between the major trans-membrane helices 2 and 3 and the other was present between the trans-membrane helices 5 and 6. These small helical regions of about ten amino acid length are of significance in the aquaporins structure since they host the filter.

Amphipathic nature of the helices was studied using hydrophobic moment sliding through the amino acid sequence of different window sizes. Tools such as EMBOSS HMOMENT (gives an overall moment of protein in the form of a plot) [25] and HeliQuest (generates a helical wheel diagram for variable windows of amino acid sequence) [26] were used to map the amphipathicity. This enabled to deduce the length of the helical regions spanning the membrane. Regions with medium hydrophobicity and high hydrophobic moment were chosen as preferred helix forming regions for all the AQPs. The results of all methods were pooled to generate secondary structure of the protein. Fig. S3 shows the optimized helical regions chosen based on both hydrophobicity and hydrophobic moment analysis. Table 1 shows the range of helical regions obtained after optimization. The second major helix of LdAQP putative was found to be the longest with 25 residues. The shortest helix were fourth and fifth major helices of LdAQP1with 18 residues (Table 1).

The topology of AQPs may not be sequentially restrained as observed in the crystal structure. However, the 3 D model of the structure can be used to understand the mechanism of both action and of passage through the channel. Hence 3 D structures were built using different templates as mentioned earlier. For model building, diverse group of representatives of AQPs were chosen based on the availability of good quality crystal structures (Table S1). Homology models were generated using EsyPred3D, 3Djigsaw and MODELLER9v8, (Fig. S4). The predicted models were compared by their RMSD values. It was observed that the RMSD values of some of the predicted models were very high. Models with least RMSD values from the templates were chosen for further analysis (Table S2a & S2b). Models built using MODELLER9v8 were found to have the lowest RMSD values consistently. Therefore only these have been shown. Figure S4 shows the alignment of the predicted structures with the templates. The trend in RMSD values (Table S2a & 2b) shows that apart from the sequence similarity shown in the phylogram, the resolution of the structural template plays an important role in building of a homology model. Hence, *P. falciparum* AQP is a better structural template than *E. coli* AQGP for building model for LdAQP1. Yeast AQP has a higher resolution of crystal structure and therefore serves as a better structural template for LdAQP2860 than *E. coli* AQP or spinach AQP. Structure of LdAQP2860 is largest in length and has the poorest RMSD due to the presence of extended N and C terminal. The overall topology and structure of different AQPs were found to be conserved. Schematic position in the membrane is shown in Fig. S5.

**Figure 3:**
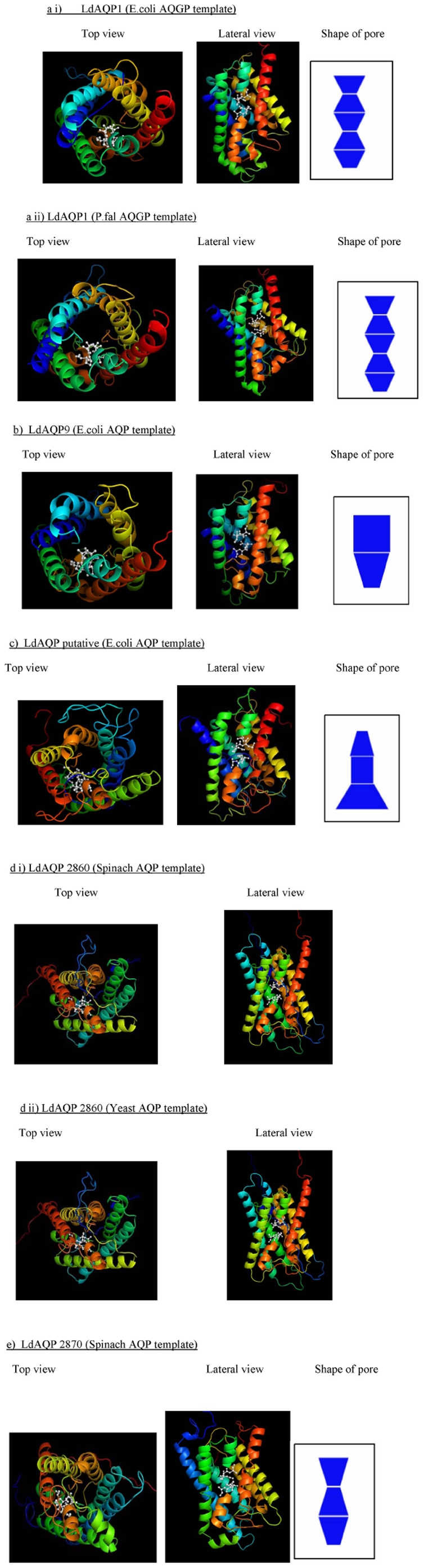
Three dimensional structures for all AQPs built using MODELLER9v8 depicting top view, lateral view and the shape of the pore in blue using PoreWalker. The position of the filter has been shown using ball and stick representation in the models for *L. donovani* AQPs a) LdAQP1 a.i) built using *E. coli* AQGP structural template, a.ii) built using *P. falciparum* AQGP structural template b) LdAQP9 built using *E. coli* AQP structural template c) LdAQP putative built using *E. coli* AQP structural template d) LdAQP2860 d.i) built using spinach AQP structural template, d.ii) built using yeast AQP structural template e) LdAQP2870 built using spinach AQP structural template. The first major transmembrane helix is shown in dark blue, second in light blue, third in bright green, fourth in yellow, fifth in light orange and sixth in red. The two small helical regions hosting the NPA motif are shown in light green (present in between major helix 2 and 3) and orange (present in between major helix 5 and 6). For LdAQP 2860 (Yeast AQP/ Spinach AQP template) i.e. for d.i), d.ii) no defined shape of the pore along the axis could be drawn. The cavity could not be defined using other tools as well.

Fig. 3 shows the three dimensional position of filters and the shape of the channel in all the built models of AQPs. LdAQP1 model was built using *P. falciparum* AQP and *E. coli* aquaglyceroporin (AQGP) template. Models of LdAQP9, LdAQP putative were built on *E. coli* AQP template. LdAQP2860 model built well using yeast AQP and spinach AQP template whereas LdAQP2870 model was built using spinach template (Fig. 3).

The MODELLER9v8 predicted structures were then validated in two stages. First, a preliminary analysis using ProSAweb and Verify3D was done (Figs. S6, S7, S8, and S9). For each of the selected models, it was found that ProSAweb showed the Z score within the range of experimentally proven (X-ray and NMR) structures. The plots show proteins having Z scores≤10, for both cytosolic and transmembrane proteins. Cytosolic proteins have Z scores in the range of +5 to −10. However, no specific range is mentioned in the case of transmembrane proteins. The largest deviation was found in LdAQP2870 model built using spinach AQP (Zscore: −3.8). The least deviation was found for LdAQP putative model built using *E.coli* AQP (Zscore: −2.53).

Verify3D showed high robustness in prediction of each amino acid forming the secondary structure. In order to visualize the dihedral angles ψ against ϕ for each residue in the given protein sequences, Ramachandran plots were made using WHAT IF [27] and MolProbity [28] (Fig. S8, S9). The Z-score and the Ramachandran map for the used templates are included in the supplementary information.

We characterized and compared the homology models built for LdAQP1, LdAQP9, LdAQP putative, LdAQP2860 and LdAQP2870. The comparison was based on the channel structure and its features containing the NPA-NPA motifs (Fig. S10). The shape & size of the tunnels were studied by various methods (Figure S11, S12, 13). Figure 3 shows the shape of these tunnels using PoreWalker [29]. PoreWalker is a novel tool for the identification and characterization of channels in transmembrane proteins from their three-dimensional structure [29]. It was observed that predicted tunnel shape in LdAQP1 built using either *E. coli* AQGP or *P. faciparum* AQP template are very similar in shape and size (Fig. 3a). These shapes are also similar to the templates shown in Fig. S13 (f & g) depicting that the channel will have the same property to transport water and glycerol like in the case of *E. coli* or *P. falciparum* AQGP. The shape of the tunnel in LdAQP9 is similar to that of *E. coli* AQP and distinctly different from that of LdAQP1 (Fig. 3b) (Fig. S13 h). LdAQP putative has a characteristic tunnel shape like that of an inverted funnel whereas LdAQP2870 has a tunnel shape partially similar to that of LdAQP1 (Fig. 3c and e). No distinct tunnel could be predicted for LdAQP2860 (Fig. 3d). This may imply that the cavity is highly irregular and undefined in geometry for this aquaporin.

The motif search using MotifScan and ScanProsite showed that LdAQP9 and LdAQP2870 have the tyrosine phoshorylation motif (Table 3). Tyrosine based motifs have been used for tracking the sub-cellular localization of certain proteins since they play a role in regulating the intracellular trafficking through trans-golgi network in certain proteins. Mutations in these regions lead to presentation of the protein on cell surface in certain proteins [30]. Motif search signifies the probability of sub-cellular localization of LdAQP9 and LdAQP2870.

**Table 3:** Summary of characterization of five AQPs of *L. donovani* studied in this report.

*L.donovani* (AQPs)	Localization	Prediction of Motif scan	Structural Model	Function	Gene Expression
AQP9	Subcellular	Tyrosine phoshorylation motif (185–192 aa)	Shape of the tunnel is similar to that of *E. coli* AQP and distinctly different from that of LdAQP1	Does not transport glycerol	Expressed
AQP- Putative	Flagellar pocket	-	Characteristic tunnel shape like that of an inverted funnel	Does not transport glycerol but transports water	Expressed
AQP2860 (Aquaporin-like protein)	ND	-	No distinct tunnel predicted; cavity is highly irregular and undefined in geometry	ND	Expression undetectable
AQP2870 (Aquaporin-like protein)	Subcellular	Tyrosine phoshorylation motif (108–115aa)	Tunnel shape partially similar to that of LdAQP1, less wider pore than all the other LdAQPs, better transporters of water than glycerol.	Does not transport glycerol	Expressed
AQP1	Posterior end	-	A bigger pore and a more hydrophobic filter	Transports water and glycerol	Expressed

The sequence obtained for AQP2870 of *L. donovani* corresponded to *AQP-like protein* LmjF.32.2380 and LinJ.32.2510 of *L. major* and *L. infantum* respectively. LdAQP2860 sequence shown in the present study for alignment and structural analysis was that of *L. infantum AQP-like protein*, LinJ.32.2500 (The Gen Bank Accession ID: XM_001467890.1).

ND: not detected.

### Localization of AQPs in *Leishmania*

**Figure 4:**
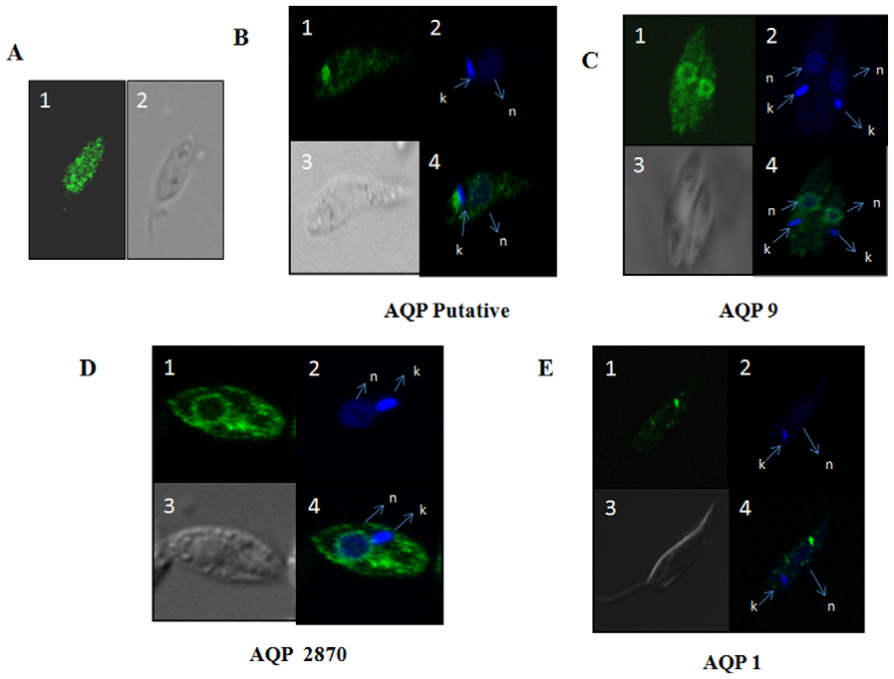
A. Localization of aquaporins in *L. donovani*. **A:** Wild type *L. donovani* transfected with pGEM7zf-αNeoα-GFP control vector expressing GFP (Panel 1). Phase contrast image (Panel 2) **B:** Confocal microscopy of wild type *L. donovani* transfected with GFP-AQP Putative as a GFP translational fusion protein (Panel 1), stained with DAPI (Panel 2), Phase contrast image (Panel 3), merged micrograph (Pane 4). **C:** Confocal microscopy of wild type *L. donovani* transfected with GFP-AQP9 as a GFP translational fusion protein (Panel 1), stained with DAPI (Panel 2), Phase contrast image (Panel 3), merged micrograph (Panel 4). **D:** Confocal microscopy of wild type *L. donovani* transfected with GFP-AQP2870 as a GFP translational fusion protein (Panel 1), stained with DAPI (Panel 2), Phase contrast image (Panel 3), merged micrograph (Panel 4). **E:** Confocal microscopy of wild type *L. donovani* transfected with GFP-AQP1 as a GFP translational fusion protein (Panel 1), stained with DAPI (Panel 2), Phase contrast image (Panel 3), merged micrograph (Panel 4).

To characterize the localization of LdAQPs, the corresponding proteins were C-terminally fused with GFP and transfected into *L. donovani* promastigotes. The parasites were allowed to grow and the localization of the AQP-GFP fusion proteins in the promastigotes was studied using confocal microscopy. Promastigotes transfected with either LdAQP9-GFP or LdAQP2870-GFP fusion proteins showed that LdAQP9 and LdAQP2870 proteins have sub-cellular localization (Figure 4 C and D). Interestingly, both LdAQP9 and LdAQP2870 were present on the nuclear membrane. Promastigotes having AQP putative -GFP fusion protein, the GFP fluorescence was limited to the anterior localization close to the flagellar pocket of the *L. donovani* promastigotes (Figure 4B). When promastigotes were transfected with AQP1-GFP, posterior localization of AQP1 was observed. This was very distinct from the anterior localization of AQP putative observed in Fig. 4B. We repeated localization studies several times and viewed approximately 90 different parasites and had consistent results. However, the parasites transfected with the GFP vector alone (lacking an insert) showed GFP fluorescence in the entire promastigote.

### Real-time PCR expression analysis of *LdAQP* genes in *L. donovani*

**Figure 5:**
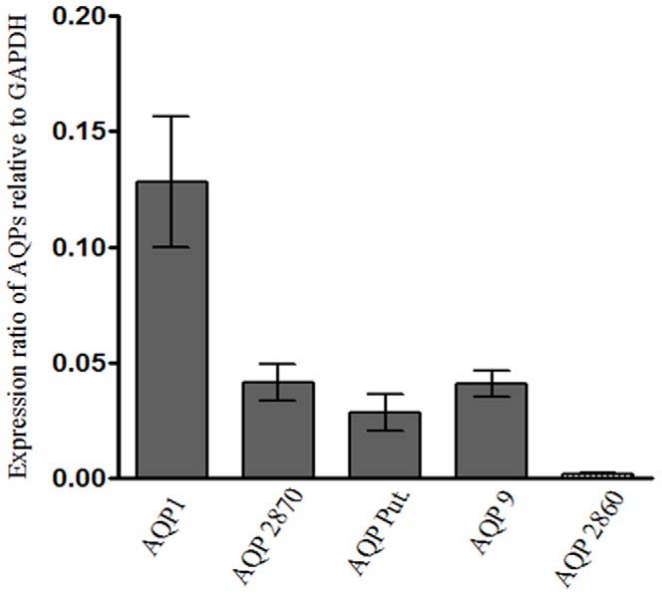
Real time RT-PCR expression analysis of AQP genes in *L. donovani*. *AQP1*, *AQP 2870*, *AQP Putative*, *AQP9* and *AQP2860* RNA expression ratios in promastigotes of *L. donovani* relative to the *GAPDH*. The graph represents mean of three independent experiments performed from three different RNA preparations. *GAPDH* gene was used for normalization.

The differential expression of the five AQP genes in the *L. donovani* strain AG83 was confirmed by real-time RT-PCR experiments (Fig. 5). The gene *AQP2860* showed undetectable levels of expression. The expression ratios of four *AQP* genes relative to the *GAPDH* are shown. Results are a mean of three independent experiments performed from three different RNA preparations. *GAPDH* gene was used for normalization.

### Cloning and heterologous expression of *L. donovani* AQPs in *S. cerevisiae*

**Figure 6:**
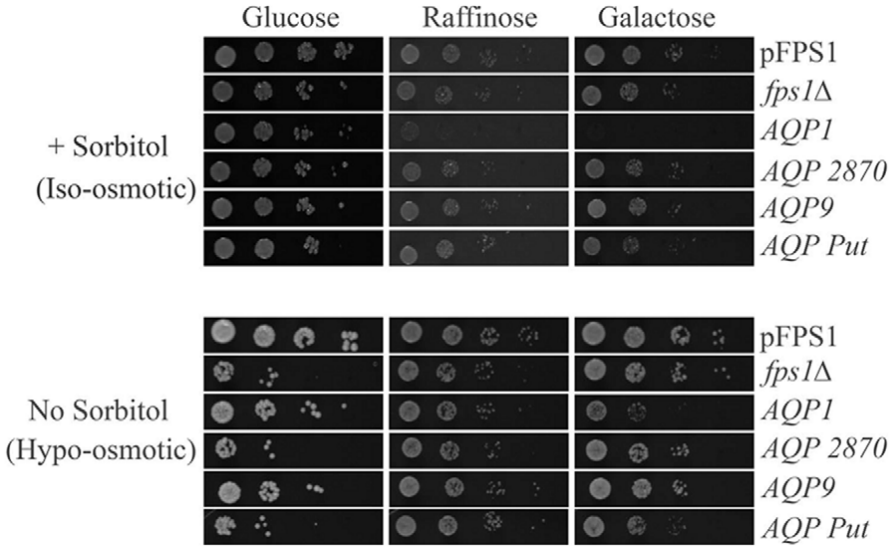
Analysis of LdAQP genes expressed in a yeast *fps1Δ* strain. Cells were cultured to saturation in selective media containing sorbitol as osmolyte and printed onto plates with or without sorbitol to study the effect of hypoosmotic shock. Cells were printed in a ten-fold dilution series either to plates containing 1 M sorbitol or onto plates with no osmolytes. Plates were incubated at 30°C for 3–5 days.

To study if the Ld*AQP* genes function as aquaglyceroporins in yeast, we cloned the *L. donovani* genes *AQP1*, *AQP2870*, *AQP9* and *AQP putative* under the regulatable yeast *GAL1* promoter (*P_GAL1_*) in the pESC-URA vector and transformed into *S. cerevisiae fps1Δ* strain. Cells were cultured in selective media containing sorbitol as osmolyte and raffinose as the carbon source and spotted to glucose, raffinose or raffinose plus galactose-containing media. Results of the growth assay showed that *fps1Δ* cells bearing *P_GAL1_*-Ld*AQP1* gene do not survive in sorbitol (isoosmotic) medium with raffinose (basal expression) or galactose (induced expression) (Fig. 6, top). The lethality is rescued when cells are printed onto plates having glucose as carbon source. This result is consistent with the unregulated glycerol channel in the *fps1Δ* strain expressing *P_GAL1_* Ld*AQP1*. Overexpression of the other *L. donovani AQP* genes did not show the same phenotype. These results indicated a glycerol channel activity for LdAQP1 in yeast. Upon hypoosmotic shock, the *AQP2870*, *AQP9* and *AQP putative* expressing strains did not show a growth defect indicating that they do not function as regulated glycerol exporters (Fig. 6).

**Figure 7:**
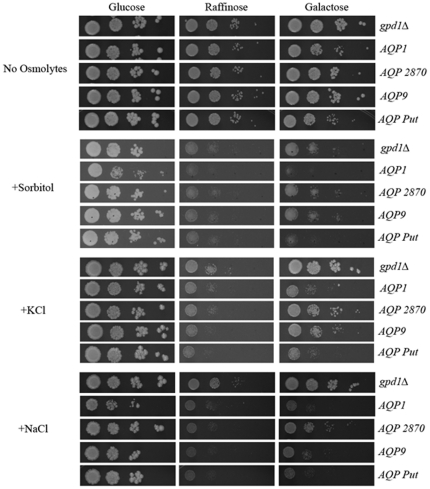
Analysis of LdAQP genes in a yeast *gpd1Δ* strain. Cells were cultured in media without osmolytes and spotted in a ten-fold dilution series onto plates supplemented with no osmolytes, 1 M Sorbitol, 0.5 M NaCl or 0.5 M KCl as indicated and incubated at 30°C for 3–5 days.

To determine the water transport activity (aquaporin function) of the cloned genes, the *P_GAL1_-AQP* plasmid constructs were transformed into YSH690 yeast strain lacking the *GPD1* gene [15]. *GPD1* codes for the enzyme glycerol-3-phosphate dehydrogenase which is involved in glycerol production, deletion of which confers osmosensitivity on yeast strains [16]. The cells were cultured in synthetic medium containing raffinose and without any osmoticum and printed onto plates containing no osmolytes (isoosmotic) or onto hyperosmotic solid media containing 1 M sorbitol, 0.5 M KCl or 0.5 M NaCl. Under isoosmotic conditions, yeast strains grew indistinguishably from those transformed with empty vector under promoter-repressing conditions (glucose medium) or under conditions of low expression (raffinose medium) (Fig. 7). The *gpd1Δ* strain expressing either Ld*AQP1* or Ld*AQP-putative* showed modest growth defect under promoter-inducing conditions (galactose medium). However on exposure to different hyperosmotic stress conditions, *gpd1Δ* cells expressing Ld*AQP1* and Ld*AQP putative* displayed a severe growth defect (Fig. 7). We also noted that *gpd1Δ* cells overexpressing Ld*AQP2870* and Ld*AQP9* displayed growth retardation relative to the control *gpd1Δ* strain transformed with empty vector. Together our data indicated that expression of Ld*AQP1* conferred glycerol as well as water transport activity, while LdAQP putative showed only water transport activity in *S. cerevisiae*.

## Discussion

Aquaglyceroporins are protein channels that transport not only water but also small uncharged solutes such as glycerol and urea [31]. These channels play important role during the various stages in the life cycle of the protozoan parasites. Parasitic protozoa are exposed to tremendous selection pressure during the transmission process between the vector and the mammalian host and tissue passages in the host blood stream [3]. These two osmotically challenging situations may require water permeability of protozoan parasite membranes. Hence, the maintenance or induction of water permeability in their aquaporin channels strongly indicates that efficient water transport is a vital property of the parasite membrane. Therefore, if this function is blocked then the parasite will die, thus making AQPs potential targets for anti-protozoan therapy. Moreover, the human and the parasite AQPs appear very different in the accessible protein structures at the pore mouth making the parasite-host interface as a prime target for chemotherapy [3].

We report here the structural modeling, localization and functional characterization of AQPs of *L. donovani*. Five AQPs have been annotated in the *L. majo*r and *L. infantum* genomes as AQP1, AQP9, AQP putative and two AQP-like proteins. LdAQP1, LdAQP9, and LdAQP putative reported in this study are identical to that reported for the *L. major* and *L. infantum* sequences. LdAQP2870 reported in this study is similar to aquaporin-like protein reported for the *L. infantum* and *L. major* sequences. LdAQP2860 sequence shown in the present study for alignment and structural analysis was that of *L. infantum AQP-like protein*, LinJ.32.2500 (The Gen Bank Accession ID: XM_001467890.1). In all the AQPs other than AQP1, carboxyl terminal to the second NPA box, a valine/alanine residue was found instead of the arginine. In that respect these AQPs are similar to tonoplast intrinsic proteins in plant, which are localized to intracellular organelles. Similarity of LdAQPs to TIPs is not surprising since kinetoplastids are known to have several plant-like genes [3].

We report here that in case of *L. donovani*, LdAQP1 is an aquaglyceroporin and shows strong similarity to *E. coli* AQGP (PDB ID: 1LDA). LdAQP1 was also found to be close to *P. falciparum* AQP and yeast AQP. LdAQP9, LdAQP putative, LdAQP2860 and LdAQP2870 in the second cluster were found to be close to each other. The nearest leaf for the known crystal structure to these was of *E. coli* AQP (2ABM).

Our localization studies of AQPs in *L. donovani* yielded some interesting results. AQP9 and AQP2870 showed sub-cellular localization in promastigotes. When promastigotes were transfected with AQP1-GFP, posterior localization of AQP1 was observed. This was very distinct from the anterior localization of AQP putative observed in Fig. 4B. Earlier studies have reported the presence of AQP1 exclusively in the flagella in *L. major*. The varied sub-cellular locations in the *L. donovani* parasites indicates that different aquaporins may have different as well as multiple physiological roles. Since *Leishmania* is a protozoan parasite and resides inside the host-macrophages, these AQPs may function as intracellular AQPs in order to survive inside the host cell. Role of intracellular aquaporins is not well defined. Interestingly, the disruption of gamma TIP has been shown to be fatal in plants [32]. Functional characterization of these intracellular AQPs in *Leishmania* would further throw light on their role in the single cell organisms.

Furthermore, aquaporins are known to function as transporters. They can be used to deliver small cytotoxic drugs inside the parasite. Certain AQPs such as AQP1 in *Leishmania* are known to transport antimonial and arsenic metalloid drugs into the parasite cytosol thereby facilitating parasite death [7]. AQP channels present promising opportunities for antiparasitic drug development. Despite being a promising drug target, knowledge about the 3D structures of many AQP proteins is somewhat limited. Recently the crystal structure of AQP in *Plasmodium falciparum* (PfAQP) was identified and analyzed to determine drug target regions [33]. Hence a great deal of work needs to be carried out in order to determine the structure, function and expression of the aquaporin proteins in protozoan parasites.

All the 5 AQP's modeled and evaluated here may be used for finding the appropriate substrate. It is worthwhile to note that LdAQP1 structure is more similar topologically to the *E. coli* AQGP than *E. coli* AQP. The topology of all the five AQPs is very similar in helix organization. However, variation in the length of the helices may result in different size and shape of the pores (Table 1). The entry of these pores may also vary as depicted in Fig. 3. This data will further help us to find the specificity of the substrate amongst the five AQPs. It was observed that the distances between the NPA-pairs (ASN-Cα) for each of the model (using PyMol) in LdAQPs to be similar (in the range of 5.18A to 5.50A). This is a requirement for functioning of the pores. It is noteworthy that glycerol transporters have wider pore than water transporters [34]. The *E. coli* AQGP [called GlpF] has a bigger pore and a more hydrophobic filter in comparison to the pure water channel (AQP) of *E. coli* [called AqpZ]. In our analysis, it was found that LdAQP2870 has a slightly less wide pore than LdAQP1, LdAQP9 and LdAQP putative. This indicates the possibility of LdAQP2870 to be better transporters of water than glycerol.

Interestingly, analyses of mRNA by Real-time PCR assays showed expression of all aquaporins except Ld*AQP2860*, whose level was undetectable. It is possible that expression of Ld*AQP2860* could be condition or stage specific in *Leishamania*. Our structural modeling studies further indicated that no distinct tunnel could be predicted for LdAQP2860. This may imply that the cavity is highly irregular and undefined in geometry for this aquaporin.

The cloning and characterization of AQP genes from *L. donovani* was done in *S.cerevisae* as an important step in determining the physiological role of these channels in the parasite. We conclude from yeast experiments that *AQP1* is an aquaglyceroporin as expression of *AQP1* conferred poor growth in sorbitol indicating glycerol loss from cells (Fig. 6). Furthermore, *AQP1* and *AQP putative* showed an osmosensitive phenotype when expressed in the *gpd1Δ* strain indicating aquaporin activity. Of these four candidate AQP genes, aquaglyceroporin activity was displayed only by the *AQP1* gene and no function could be ascribed to *AQP9* and *AQP2870* using the yeast model system. Intracellular localization of AQP9 and AQP2870 could be one of the reasons that preclude the functional analysis by conventional methods. Currently, little is known about the function of intracellular AQPs.

In conclusion, we report structural modeling, localization and functional characterization of AQPs from *L. donovani* (Table 3). We for the first time show the presence of subcellular-aquaporins in *L. donovani* that are similar to tonoplast intrinsic protein of plants. Our analyses provided structural and functional basis for the identification of *L. donovani AQP1* as aquaglyceroporin and Ld*AQP1* and Ld*AQP putative* as aquaporins. Further studies are underway to improve our understanding of these transporters and clarify the role of the intracellular AQPs.

## Materials and Methods

### Materials

All restriction enzymes and DNA modifying enzymes were obtained from MBI Fermentas (Germany). Cloning vector pTZ75R/T was obtained from MBI Fermentas (Germany) and yeast epitope tagging vector pESC-URA (Stratagene, CA, USA) was used for cloning and heterologous expression. Vectors pGEM-7zf-aNeoα-GFP and pSP72-αNeoα-GFP (containing neomycin phosphotransferase as the selection marker) were provided by Dr. Marc Ouellette (University of Laval, Quebec, Canada). Neomycin (G418) was obtained from Sigma Aldrich Corp. (St. Louis, MO). The other materials used in this study were of the highest purity and were commercially available.

### Cell Culture

*L. donovani* promastigote (MHOM/IN/80/AG83) were grown at 22°C in M199 medium (Sigma St. Louis, MO) supplemented with 100 units/ml penicillin (Sigma, St. Louis, MO), 100 µg/ml streptomycin (Sigma, St. Louis, MO) and 10% fetal bovine serum (FBS, Hyclone, U.K.). Yeast strains used in this study were W303-1A (*MATa leu2-3 leu2-112 ura3-1 trp1-1 his3-11 his3-15 ade2-1can1-100 GAL SUC2 mal0*), YSH294 (*MATa leu2-3 leu2-112 ura3-1 trp1-1 his3-11 his3-15 ade2-1 can1-100 GAL SUC2 fps1Δ:LEU2*) and YSH690 (*MATa leu2-3 leu2-112 ura3-1 trp1-1 his3-11 his3-15 ade2-1 can1-100 GAL SUC2 mal0 gpd1Δ::TRP1*) were provided by Dr. Stefan Hohmann (Goteborg University, Sweden). Cells were grown on synthetic minimal medium (1.45 g yeast nitrogen base without amino acids and ammonium sulfate (BD-DIFCO), 5 g ammonium sulfate, 20 g Bacto-Agar (BD-DIFCO) each per liter), and amino acids as described previously [35] and containing 2% (w/v) glucose. For the selection of transformants, cells were plated on synthetic minimal medium lacking leucine. Phenotype was analyzed by growth assay on indicated plates.

### Cloning of aquaporin genes from *L. donovani* and heterologous expression in *S. cerevisiae*

Cloning was performed for four *Leishmania donovani* aquaporins using specific oligonucleotides whose sequence was based on *Leishmania* Genome Sequencing Project of *L. infantum* (http://www.ebi.ac.uk/parasites/LGN/). AQP genes were amplified from the genomic DNA of *L. donovani* strain AG83. A list of primers and PCR conditions used for each gene is provided in Table S3a. PCR products were cloned into pTZ75R/T (MBI, Fermentas) and sequenced. The PCR products were also sub-cloned into pESC-URA (yeast expression vector) using *Bam*HI and *Sal*I restriction sites.

### Functional analysis of aquaporins in yeast heterologous system

The *S. cerevisiae* strains YSH294 and W303-1A [9] were used in the present study. The strain YSH294 lacks the yeast aquaglyceroporin *FPS1* gene. The four *L. donovani AQP* genes, the empty vector control pESC-URA and pFPS1 containing *FPS1* gene bearing native promoter were transformed into the strain YSH294 [36]. The strain W303-1A was transformed with empty vector pESC-URA and used as wild-type control. For determination of glycerol transport activity, different strains were cultured in selective SC medium plus 1 M sorbitol and 2% raffinose as the carbon source for 24 hours. Ten-fold serial dilutions of the strains were spotted onto SC medium plus 1 M sorbitol containing 2% glucose, 2% raffinose, or 1% raffinose and 1% galactose as carbon sources.

For determination of water transport activity, the plasmid constructs for all four Ld*AQP* genes were transformed to the yeast strain YSH690 [15], containing a deletion in the *GPD1* gene involved in glycerol production. As controls, W303-1A and YSH690 were transformed with empty vector pESC-URA as positive and negative controls respectively. The strains were cultured in selective SC media containing 2% raffinose as carbon source and spotted onto plates with or without 1 M sorbitol, 0.5 M NaCl or 0.5 M KCl in presence of glucose, raffinose, or galactose and raffinose as the carbon source. The plates were buffered with 10 mM Na-succinate and pH adjusted to 6.5 [15].

### Construction of AQP-GFP fusion constructs and transfection

The LdAQP genes were amplified from the genomic DNA of *L. donovani* strain AG83 by using gene specific primers. A list of primers and PCR conditions used for each gene is provided in Table S3b. The amplified gene products were cloned into the GFP containing vector pSP-72zαneoα-GFP or pGEM-7zfαNeoα. The clones were checked for correct orientation of the insert by restriction enzyme digestion and confirmed by sequencing. 20 µg of the plasmid was transfected into the *L. donovani* promastigotes (MHOM/IN/80/AG83). Methods of electroporation and plating of *L. donovani* have been described previously [37]. The transfected cells were maintained in 40 µg/ml of G418. These transfected parasites were used for localization studies. A control transfection with vector alone in to the *L. donovani* parasites was used for comparison.

### Localization of Aquaporins

To detect the site of localization of these aquaporins in *L. donovani*, the GFP-transfected promastigotes were used. Fluorescent imaging of the stabilized culture was performed using the confocal laser scanning microscope (Zeiss LSM 510 META) equipped with a 63× objective, at an excitation wavelength of 488 nm. Briefly 10^7^ promastigotes/ml were pelleted. The cells were then washed with phosphate-buffered saline (PBS) containing 1% fetal bovine serum (FBS) and resuspended in the same PBS solution with identical final cell concentration. The promastigotes were then immobilized on poly (L) lysine coated cover-slips. The cover-slips were incubated in ice-cold paraformaldehyde for 20 min followed by washing with PBS. Cells were then permeabilized using 0.5% Triton-X. Transfected promastigotes were stained with DAPI (0.1 µg/ml) for 15 min at room temperature. The parasites stained with DAPI were observed at an excitation wavelength of 405 nm.

### RNA preparation and real-time PCR (RT-PCR)

*Leishmania* total RNA was isolated using RNAeasy kit (Quiagen, Germany), treated with DNAse. Reverse transcription (RT) was performed according to manufacturer instructions using First-strand cDNA synthesis kit (Fermentas, Germany) in a 20 µl reaction containing 500 ng purified RNA. Controls containing the same amount of RNA but lacking reverse transcriptase or template were used to rule out DNA or other contamination. Primers used for individual aquaporins are shown in Table S3c. PCRs were performed using the SYBR Green (Applied Biosystems) and the ABI PRISM 7000 Sequence Detection System instrument (Applied Biosystems). PCR amplifications were performed as follows: 50°C for 2 min and 95°C for 10 sec followed by 40 cycles at 95°C for 30 sec, 62°C for one min and 72°C for 20 sec. The generation of specific PCR products was confirmed by melting curve analysis. All samples were performed in triplicates. Amplification of Glyceraldehyde -3-phosphate dehydrogenase (GAPDH) was used as an internal control.

### Comparative modeling of *L. donovani* aquaporins

Generating a homology model included identification of structural template(s), alignment of target sequence and template structure(s), model building and model quality evaluation.

### Sequence alignment and phylogeny

For comparative analysis, *L. donovani* AQP protein sequences were aligned with AQPs from various organisms with known crystal structures. Phylogenetic relationship was deduced using a Phylogeny.fr program [38] in ‘advance mode’. In this mode, multiple sequence alignment was done using MUSCLE and curation was done using G block program. This helped to eliminate poorly aligned positions and divergent regions. Phylogeny was built using PhyML program and tree was rendered by TreeDyn [38]. Due to the distinct symmetry of the six helices forming an hour-glass structure, the sequence of each of the helix bundles (TM1, TM2, TM3 and TM4, TM5, TM6) were aligned with *L. major* (All 5 AQPs) and *L. donovani* (All 5 AQPs) sequences. The sequences were also aligned with the other sequences for which the crystal structures were available like human AQP1 [PDB ID: 1IH5], yeast [PDB ID: 2W2E], rat [PDB ID: 2D57], spinach [PDB ID: 1Z98], *P.falciparum* [PDB ID: 3C02], *E.coli* aquaporin [PDB ID: 2ABM] and *E.coli* aquaglyceroporin [PDB ID: 1LDA] [39]–[45].

### Role of hydrophobicity and hydrophobic moment to identify the six helices

It was difficult to use the whole sequence for multiple sequence alignment in case of *L. donovani* AQPs since the length of the protein sequences varied from 230 (in LdAQP9) to 596 (in LdAQP 2860) amino acids. Hence, we compared the helix forming transmembrane regions and the NPA motif containing region of all the AQPs. All AQPs shared the same core topology of the six major transmembrane helical regions and the two minor helices hosting the NPA motifs. Thereafter, the hydrophobicity and hydrophobic moments were used to obtain the length of the helical regions in each of the AQP protein sequence. For each of the sequences, hydropathy plots were generated using TMHMM server 2.0, ProtScale and OCTOPUS [23], [24]. The hydrophobic regions were then identified for amphipathicity by plotting HMOMENT plot by Hmoment [46]. and plotting helical wheels using HeliQuest [26]. Using these programs, the conservative sequence range was identified to consider the helix bundle formation.

### Generation of homology model

Using different templates, homologous structures were built by three different programs EsyPred3D, 3Djigsaw and MODELLER9v8 [47]–[49]. Models generated from these were compared based on their deviation in terms of RMSD from the used template. PyMol [50] was used for RMS calculation [51]. It was observed that MODELLER9v8 produced models with consistent low RMS deviations with respect to the template. Hence, structure quality evaluation was done for these models.

Homology models were generated for *L. donovani* AQPs by comparing the transmembrane region domains of the nearest neighbors in the phylogram. Based on the phylogeny, LdAQP1 was found to be nearest to the *E. coli* aquaglyceroporin AQGP [PDB ID: 1LDA, chain A, resolution 2.8A°]. Hence, *E. coli* aquaglyceroporin AQGP was used to build the structure of the LdAQP1. *P. falciparum* AQP [PDB ID: 3C02, chain A, resolution 2.05A°] structural template was also used as a template for modeling LdAQP1. This was keeping in view the fact that it is better in quality in terms of resolution and is one of the near neighbors of LdAQP1. LdAQP9, putative, 2860 and 2870 showed strong relatedness to *E.coli* AQP crystal structure [PDB ID: 2ABM, chain A, resolution: 3.2A°]. Hence, *E.coli* AQP crystal structure was used to build the structure of LdAQP9, putative, 2860 and 2870. The next close neighbor of these three LdAQPs was spinach [PDB ID: 1Z98, chain A, resolution: 2.10 A°]. This structure was also used for modeling these three LdAQPs. Yeast AQP crystal structure [PDB ID: 2W2E, chain A, resolution of 1.15A°] due to its high resolution was used as a structural template for modeling *LdAQP2860*.

### Validation of the predicted model

Each of the models built was validated using ProSAweb [52]. This showed that the Z score for these models was within the range of experimentally proven X-ray and NMR structures. Verify3D [53] was also used for validation of the built models. This method showed the accuracy in prediction of each amino acid at a position forming associated secondary structure. Both these validation methods were preliminary in nature. Hence Ramachandran plots (phi, psi angles) were drawn for these models using WHAT IF [27] and MolProbity [28].

## Supporting Information

Figure S1 **Multiple sequence alignment for complete amino acid sequences of AQPs using ClustalW.** The small (small+hydrophobic (incl.aromatic -Y)) have been marked in RED, acidic residues in BLUE, basic residues in MAGENTA, GREEN marks Hydroxyl+Amine+Basic - Q and others in Gray. “*” means that the residues or nucleotides in that column are identical in all sequences in the alignment. “:” means that conserved substitutions have been observed, according to the COLOUR table above. “.” means that semi-conserved substitutions are observed. NPA mtoif has been highlighted with yellow, however, non canonical motifs in filter have been marked in green. The NCBI accession numbers of the protein sequences are as follows: LdAQP1: gi|148533557.1, LmAQP1: gi|68128057, human AQP9: gi|2887407, 1LDA_*E. coli* AQGP: gi|21466052, 3C02_*P. falciparum*: gi|189096170, 1IH5_human1: gi|14278358, 2D57_Rat: gi|88192744, 1Z98_Spinach: gi|85544014, LdAQP9: gi|269854619, LmAQP9: gi|68129565, Ld2860: gi|146096773 LmAQP2860: gi|157874137, Ld_put: gi|269854615, LmAQP_p: gi|68224177, LdAQP2870: gi|269854616 LmAQP2870: gi|157874135, 2ABM_*E. coli*AQP: gi|78101284, 2W2E_Yeast: gi|240104254.
(DOCX)

Figure S2 (1) TMHMM server result for *L. donovani* AQPs, LdAQP1 (a), LdAQP9 (b), LdAQP putative (c), LdAQP2860 (d), LdAQP2870 (e). Marked in red are transmembrane regions, while loops present on the interior are marked in blue and loops present on the exterior side are marked in magenta. Six distinct transmembrane regions and two small regions between major TM 2–3 and TM 5–6 were predicted. (2) OCTOPUS predicted topology *for L. donovani AQPs*, LdAQP1 (a), LdAQP9 (b), LdAQP putative (c), LdAQP2860 (d), LdAQP2870 (e). The three panels in each figure show i) topology of the protein with six major transmembrane regions ii) six major and two minor transmembrane regions marker in red iii) regions present internal and external to the membrane.
(DOCX)

Figure S3 **HeliQuest output for** ***L. donovani*** **.** AQPs a) LdAQP1 b) LdAQP9, c) LdAQP putative d) LdAQP2860 e) LdAQP2870. Regions 1 to 6 have been shown in the form of helical wheel from A to F respectively. The best helical wheels, with medium hydrophobicity and high hydrophobic moment have been shown for each transmembrane region. Yellow region shows the hydrophobic face of helix.
(DOCX)

Figure S4 Structure predicted for *L. donovani* AQPs by A. EsyPred3D, B. 3D Jigsaw, C. MODELLER9v8. D. All three predicted models aligned with template. a) LdAQP1 a.i.) Template: *E. coli* AQGP [PDB ID: 1LDA, Resolution: 2.8 A°] a.ii.) Template: *P. falciparum* AQP* [PDB ID: 3C02, Resolution: 2.05°] b) LdAQP9 [Template: *E.coli* AQP, PDB ID: 2ABM, Resolution: 3.2 A°], c) LdAQP putative [Template: *E. coli* AQP, PDB ID: 2ABM, Resolution: 3.2 A°] d) LdAQP2860 d.i.) Template: Spinach AQP* [PDB ID: 1Z98, Resolution: 2.10 A°] and d.ii.) Template: Yeast AQP* [PDB ID: 2W2E, Resolution: 1.15 A°] e) LdAQP2870 [Template: Spinach AQP, PDB ID: 1Z98, Resolution: 2.10 A°]. *For these sequences the 3Djigsaw prediction was not possible due to poor sequence alignment. Hence the third image in a.ii), d.i), d.ii) are the alignment of both predicted models over the template.
(DOCX)

Figure S5 **The 2 D topology of predicted structure for** ***Leishmania donovani*** **AQPs.** *a)* LdAQP1 b) LdAQP9, c) LdAQP putative d) LdAQP2860 e) LdAQP2870, showing the six major transmembrane helices and two small helices.
(DOCX)

Figure S6 **ProSAweb output for** ***L. donovani*** **AQPs predicted using MODELLER9v8 has been marked with a black dot.** a) LdAQP1 a.i.) built using template *E. coli* AGP [*Z*-Score: −2.59] a.ii.) built using template *P. falciparum* AQP [Z-score: −2.60] b) LdAQP9 built using *E. coli* AQP [*Z*-Score: −2.84], c) LdAQP putative built using *E. coli* AQP [*Z*-Score: −2.53] d) LdAQP 2860 d.i) built using spinach AQP [Z-score: −3.61] d.ii.) built using yeast AQP [*Z*-Score: −3.45] e) LdAQP 2870 built using spinach AQP [*Z*-Score: −3.81]. In addition to the predicted models, the Z scores obtained for the templates are also shown. f) *E. coli* AQGP [PDB ID: 1LDA,*Z*-Score: −5.61] g) *P. falciparum* AQP [PDB ID: 3C02, *Z*-Score: −4.37] h) *E. coli* AQP [PDB ID: 2ABM, *Z*-Score: −4.55] i) Yeast AQP [PDB ID: 2W2E, *Z*-Score: −6.54] j) Spinach AQP [PDB ID: 1Z98, *Z*-Score: −4.36]. ProSAweb z-scores of all protein chains in PDB determined by X-ray crystallography are shown in light blue, whereas, those derived using NMR spectroscopy are shown in dark blue.
(DOCX)

Figure S7 Verify3D output for *L. donovani* AQPs predicted using MODELLER9v8, a) LdAQP1 a.i.) built using template *E. coli* AGP a.ii.) built using template *P. falciparum* AQP b) LdAQP9 built using *E. coli* AQP c) LdAQP putative built using *E. coli* AQP d) LdAQP 2860 d.i) built using spinach AQP d.ii.) built using yeast AQP e) LdAQP 2870 built using spinach AQP. In addition to the predicted models, the plots obtained for the templates are also shown. f) *E. coli* AQGP [PDB ID: 1LDA] g) *P. falciparum* AQP [PDB ID: 3C02] h) *E. coli* AQP [PDB ID: 2ABM] i) Yeast AQP [PDB ID: 2W2E] j) Spinach AQP [PDB ID: 1Z98]. The score (accuracy in prediction) for the full sequence has been shown on Y axis, however, X axis has residue numbers. Green line marks the highest score, while orange line denotes the zero score, and purple shows lowest score in prediction.
(DOCX)

Figure S8 Validation of the MODELLER9v8 predicted structure using WHAT IF for *L. donovani* AQPs, a) LdAQP1 a.i.) built using template *E. coli* AGP a.ii.) built using template *P. falciparum* AQP b) LdAQP9 built using *E. coli* AQP c) LdAQP putative built using *E. coli* AQP d) LdAQP2860 d.i) built using spinach AQP d.ii.) built using yeast AQP e) LdAQP2870 built using spinach AQP. In addition to the predicted models, the plots obtained for the templates are also shown. f) *E. coli* AQGP [PDB ID: 1LDA] g) *P. falciparum* AQP [PDB ID: 3C02] h) *E. coli* AQP [PDB ID: 2ABM] i) Yeast AQP [PDB ID: 2W2E] j) Spinach AQP [PDB ID: 1Z98]. Here, blue means helix, red means strand and green means turn and loop (according to DSSP). The lines in the plot indicate prefered areas. The outer lines encircle the area within which 90% of all crosses of the same colour should be found; the inner lines indicate the 50% area. Orthogonal crosses indicate ‘normal’ residues; diagonal crosses indicate glycines and open squares indicate prolines.
(DOCX)

Figure S9 Validation of the MODELLER9v8 structures using MolProbity for *L. donovani* AQPs, a) LdAQP1 a.i.) built using template *E. coli* AGP a.ii.) built using template *P. falciparum* AQP b) LdAQP9 built using *E. coli* AQP c) LdAQP putative built using *E. coli* AQP d) LdAQP2860 d.i) built using spinach AQP d.ii.) built using yeast AQP e) LdAQP2870 built using spinach AQP. In addition to the predicted models, the plots obtained for the templates are also shown. f) *E. coli* AQGP [PDB ID: 1LDA] g) *P. falciparum* AQP [PDB ID: 3C02] h) *E. coli* AQP [PDB ID: 2ABM] i) Yeast AQP [PDB ID: 2W2E] j) Spinach AQP [PDB ID: 1Z98]. Ramachandran plot was obtained showing allowed/correct conformations in the predicted model. This analysis also gave the residue numbers that have incorrect conformations as outliers.
(DOCX)

Figure S10 Top-down view of MODELLER9v8 structures for *L. donovani* AQPs, LdAQP1 a.i.) built using template *E. coli* AGP a.ii.) built using template *P. falciparum* AQP b) LdAQP9 built using *E. coli* AQP c) LdAQP putative built using *E. coli* AQP d) LdAQP2860 d.i) built using spinach AQP d.ii.) built using yeast AQP e) LdAQP2870 built using spinach AQP. The first major transmembrane helix in shown in dark blue, second in light blue, third in bright green, fourth in yellow, fifth in light orange and sixth in red. The two small helical regions hosting the NPA motif are shown in light green (present in between major helix 2 and 3) and orange (present in between major helix 5 and 6).
(DOCX)

Figure S11 **Porewalker predicted pore diameter at 3 A° in LdAQPs.** a) LdAQP1 a.i.) built using template *E. coli* AQGP a.ii.) built using template *P. falciparum* AQP b) LdAQP9 built using *E. coli* AQP c) LdAQP putative built using *E.coli* AQP d) LdAQP2860 d.i) built using spinach AQP d.ii.) built using yeast AQP e) LdAQP2870 built using spinach AQP. In addition to the predicted models, the plots obtained for the templates are also shown. f) *E. coli* AQGP [PDB ID: 1LDA] g) *P. falciparum* AQP [PDB ID: 3C02] h) *E. coli* AQP [PDB ID: 2ABM] i) Yeast AQP [PDB ID: 2W2E] j) Spinach AQP [PDB ID: 1Z98]. PoreWalker is unable to handle too long queries, as in case of d.i), d.ii).
(DOCX)

Figure S12 **PoreWalker predicted pore orientation in LdAQPs.** a) LdAQP1 a.i.) built using template *E. coli* AQGP a.ii.) built using template *P. falciparum* AQP b) LdAQP9 built using *E. coli* AQP c) LdAQP putative built using *E. coli* AQP d) LdAQP 2860 d.i) built using spinach AQP d.ii.) built using yeast AQP e) LdAQP2870 built using spinach AQP. In addition to the predicted models, the plots obtained for the templates are also shown. f) *E. coli* AQGP [PDB ID: 1LDA] g) *P. falciparum* AQP [PDB ID: 3C02] h) *E. coli* AQP [PDB ID: 2ABM] i) Yeast AQP [PDB ID: 2W2E] j) Spinach AQP [PDB ID: 1Z98]. PoreWalker is unable to handle too long queries, as in case of d.i), d.ii).
(DOCX)

Figure S13 **PoreWalker predicted tunnel shape in LdAQPs.** a) LdAQP1 a.i.) built using template *E. coli* AQGP a.ii.) built using template *P. falciparum* AQP b) LdAQP9 built using *E. coli* AQP c) LdAQP putative built using *E. coli* AQP d) LdAQP 2860 d.i) built using spinach AQP d.ii.) built using yeast AQP e) LdAQP 2870 built using spinach AQP. In addition to the predicted models, the plots obtained for the templates are also shown. f) *E. coli* AQGP [PDB ID: 1LDA] g) *P. falciparum* AQP [PDB ID: 3C02] h) *E.coli* AQP [PDB ID: 2ABM] i) Yeast AQP [PDB ID: 2W2E] j) Spinach AQP [PDB ID: 1Z98]. PoreWalker is unable to handle too long queries, as in case of d.i), d.ii).
(DOCX)

Table S1 **AQPs from different species with reported crystal structures.**
(DOCX)

Table S2 (a) RMSD values for models predicted using MODELLER9v8 for all *L. donovani* AQPs with structural templates: *P. falciparum* [PDB ID: 3C02, chain A, Resolution: 2.05 A°], *E. coli* AQGP [PDB ID: 1LDA, chain A, Resolution: 2.8 A°], *E. coli* AQP [PDB ID: 2ABM, chain A, Resolution: 3.2 A°], Spinach AQP [PDB ID: 1Z98, chain A, Resolution: 2.10 A°], Yeast AQP [PDB ID: 2W2E, chain A, Resolution: 1.15 A°]. b: RMSD values for all models predicted using EsyPred3D, 3Djigsaw and MODELLER9v8 for five *L. donovani* AQPs with various structural templates: *P. falciparum* [PDB ID: 3C02, chain A, Resolution: 2.05 A°], *E. coli* AQGP [PDB ID: 1LDA, chain A, Resolution: 2.8 A°], *E. coli* AQP [PDB ID: 2ABM, chain A, Resolution: 3.2 A°], Spinach AQP [PDB ID: 1Z98, chain A, Resolution: 2.10 A°], Yeast AQP [PDB ID: 2W2E, chain A, Resolution: 1.15 A°].
(DOCX)

Table S3 (A) List of Primers used (restriction enzyme sites underlined) for getting PCR products that were cloned into pESC-URA (yeast expression vector). b: List of Primers used (restriction enzyme sites underlined) for getting PCR products that were cloned into Leishmania specific-GFP vectors c: List of Primers used for Real time PCR.
(DOCX)
