# Supplementary figures and images for: Characterization of Leishmania donovani Aquaporins Shows Presence of Subcellular Aquaporins Similar to Tonoplast Intrinsic Proteins of Plants
Source: PLoS One. 2011 Sep 28;6(9):e24820. doi: 10.1371/journal.pone.0024820 (PMC3182166; doi:10.1371/journal.pone.0024820)

**Figure S1**


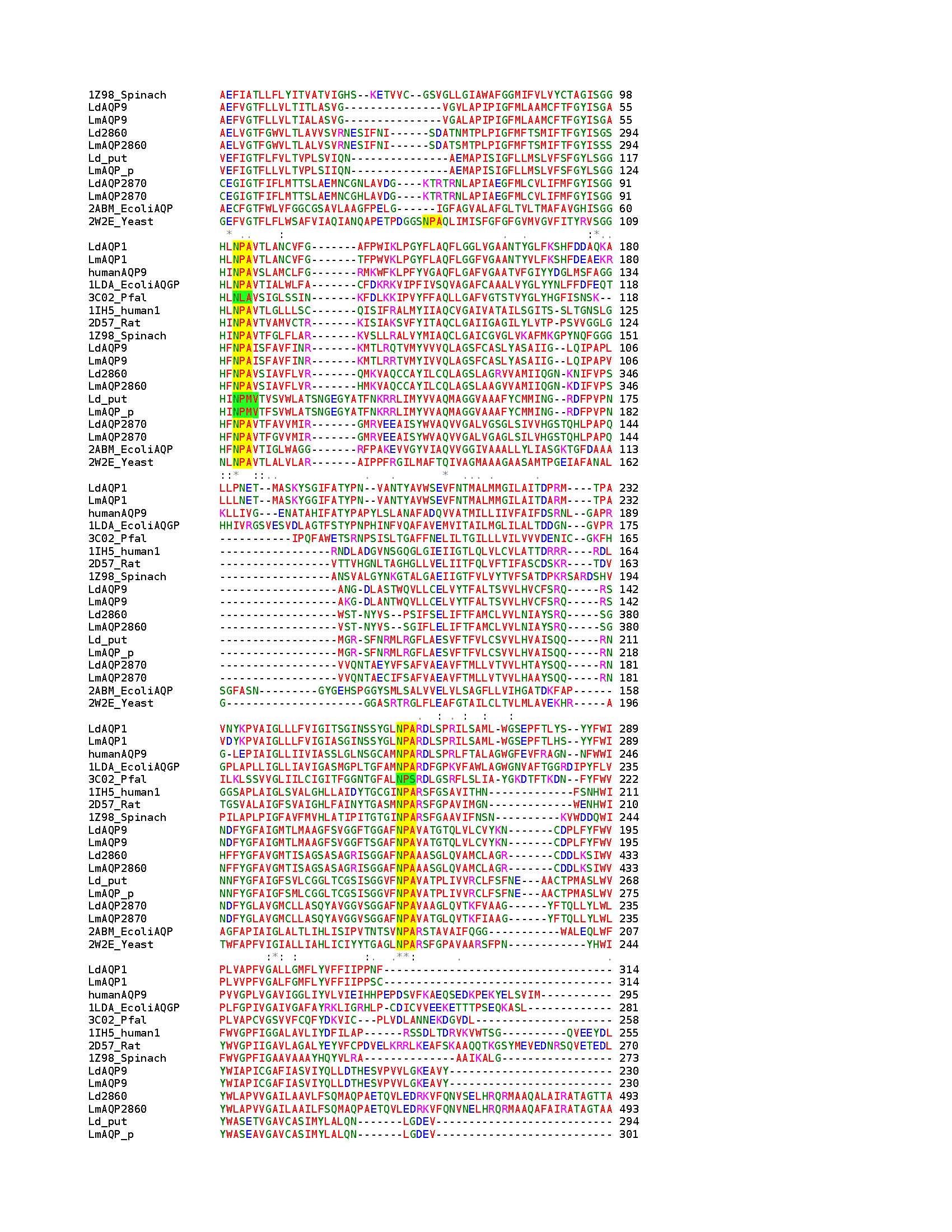


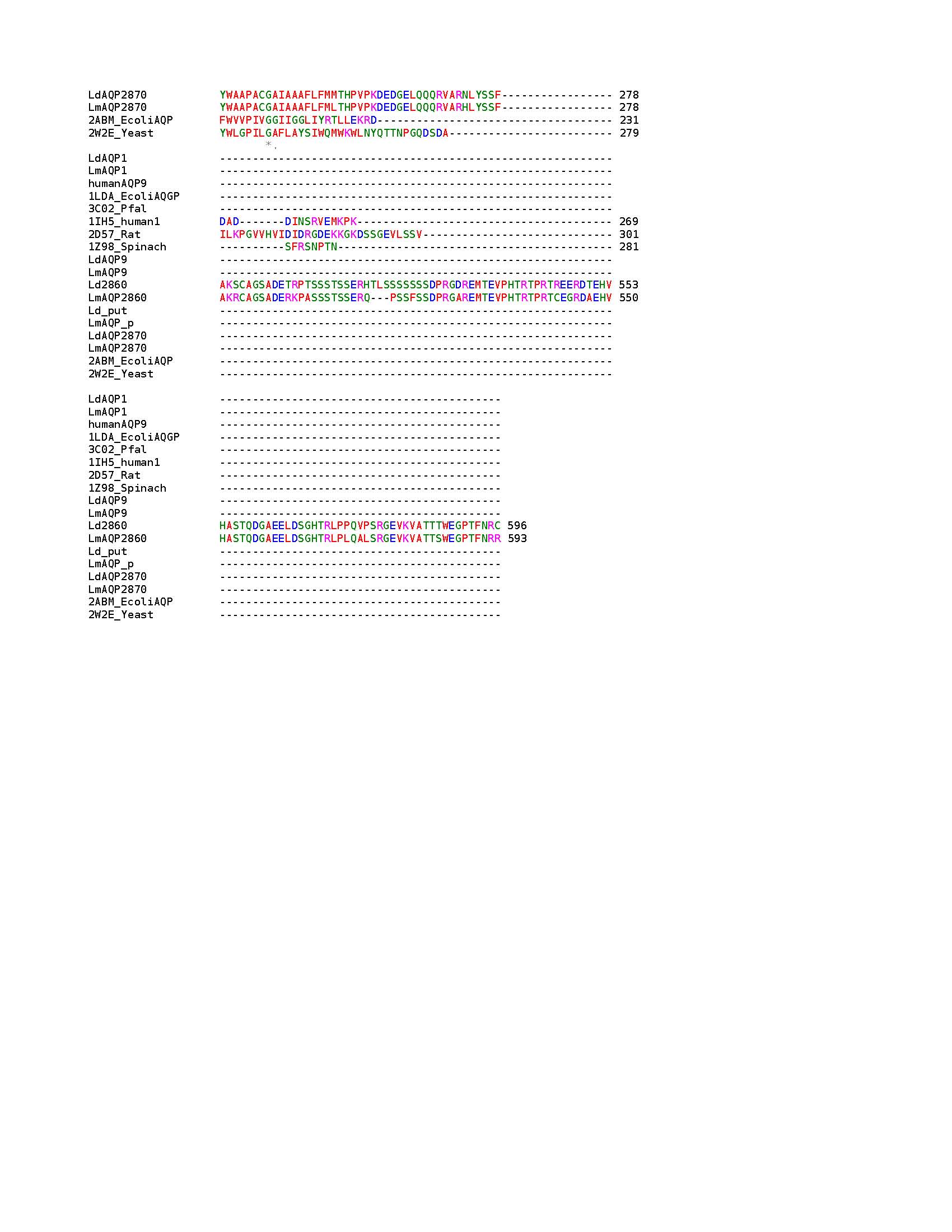

Supplement: Figure S1 — Multiple sequence alignment for complete amino acid sequences of AQPs using ClustalW. The small (small+hydrophobic (incl.aromatic -Y)) have been marked in RED, acidic residues in BLUE, basic residues in MAGENTA, GREEN marks Hydroxyl+Amine+Basic - Q and others in Gray. “*” means that the residues or nucleotides in that column are identical in all sequences in the alignment. “:” means that conserved substitutions have been observed, according to the COLOUR table above. “.” means that semi-conserved substitutions are observed. NPA mtoif has been highlighted with yellow, however, non canonical motifs in filter have been marked in green. The NCBI accession numbers of the protein sequences are as follows: LdAQP1: gi|148533557.1, LmAQP1: gi|68128057, human AQP9: gi|2887407, 1LDA_E. coli AQGP: gi|21466052, 3C02_P. falciparum: gi|189096170, 1IH5_human1: gi|14278358, 2D57_Rat: gi|88192744, 1Z98_Spinach: gi|85544014, LdAQP9: gi|269854619, LmAQP9: gi|68129565, Ld2860: gi|146096773 LmAQP2860: gi|157874137, Ld_put: gi|269854615, LmAQP_p: gi|68224177, LdAQP2870: gi|269854616 LmAQP2870: gi|157874135, 2ABM_E. coliAQP: gi|78101284, 2W2E_Yeast: gi|240104254. (DOCX) [file pone.0024820.s001.docx]

**Figure S2** (**1)**

a)


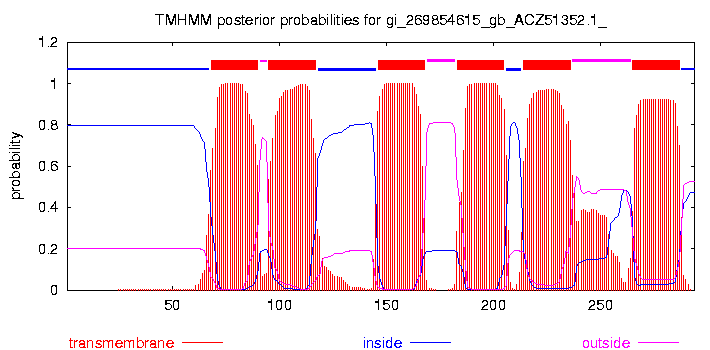


b)


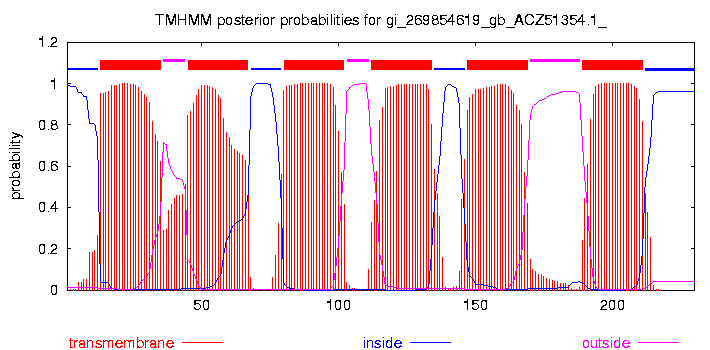


c)


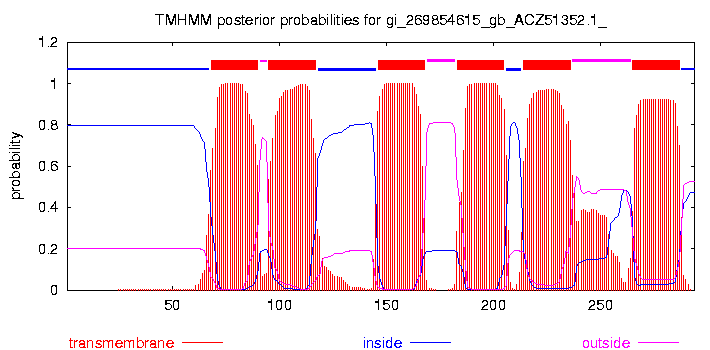


d)


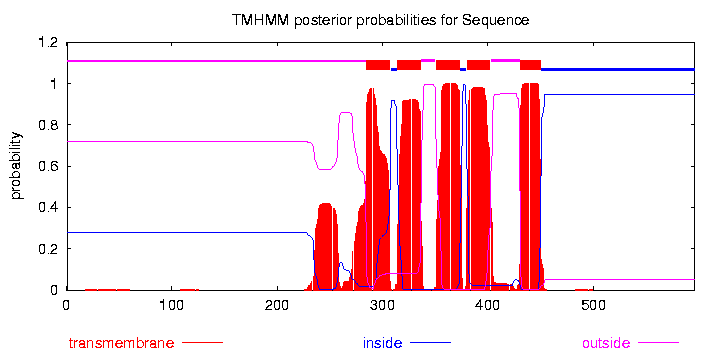


e)


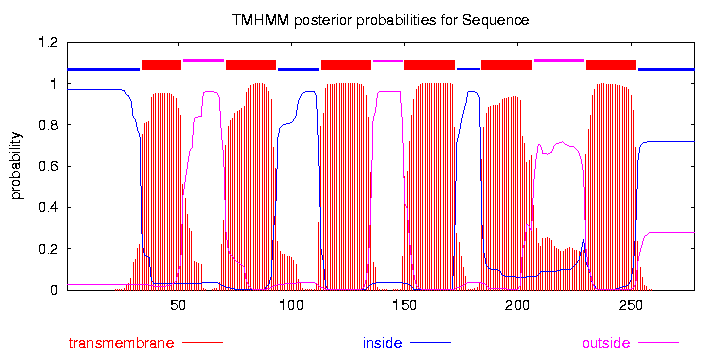


**Figure S2** **(2**)

a)


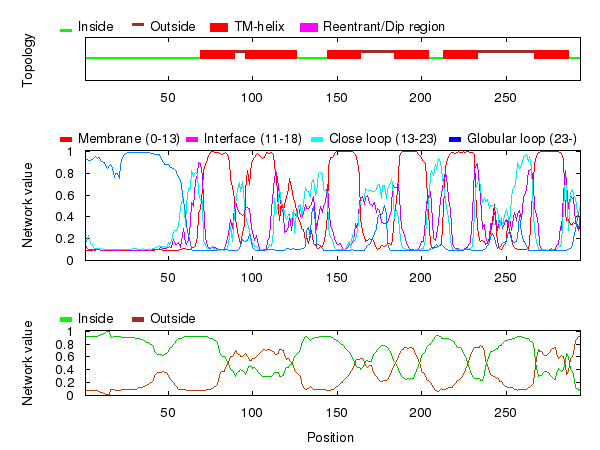


b)


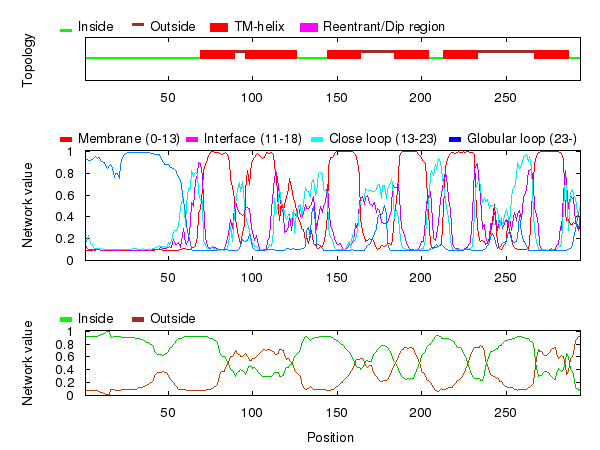
c)


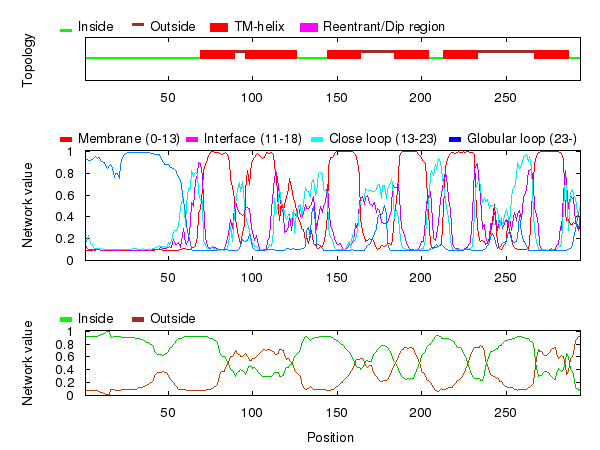


d)


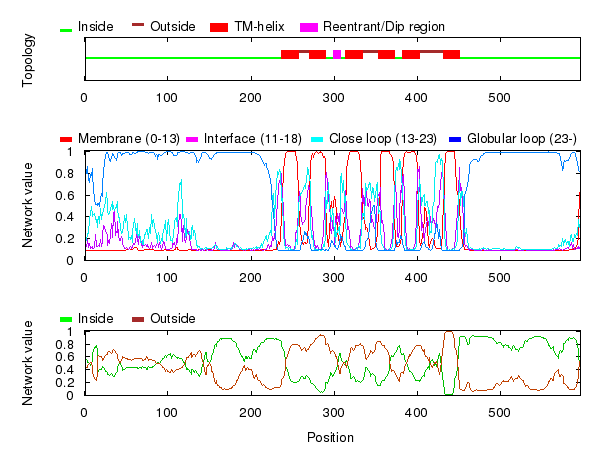


e)


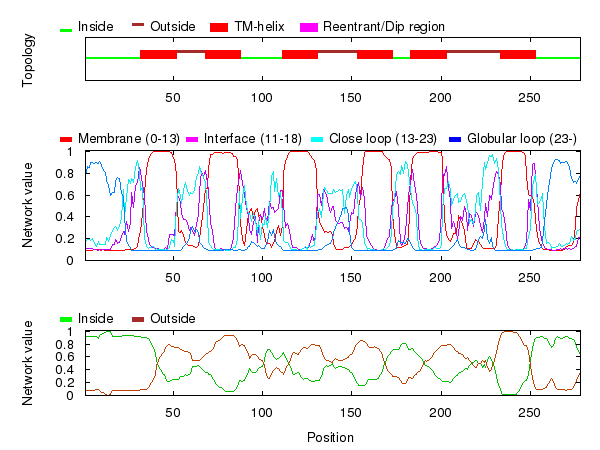

Supplement: Figure S2 — (1) TMHMM server result for L. donovani AQPs, LdAQP1 (a), LdAQP9 (b), LdAQP putative (c), LdAQP2860 (d), LdAQP2870 (e). Marked in red are transmembrane regions, while loops present on the interior are marked in blue and loops present on the exterior side are marked in magenta. Six distinct transmembrane regions and two small regions between major TM 2–3 and TM 5–6 were predicted. (2) OCTOPUS predicted topology for L. donovani AQPs, LdAQP1 (a), LdAQP9 (b), LdAQP putative (c), LdAQP2860 (d), LdAQP2870 (e). The three panels in each figure show i) topology of the protein with six major transmembrane regions ii) six major and two minor transmembrane regions marker in red iii) regions present internal and external to the membrane. (DOCX) [file pone.0024820.s002.docx]

**Figure S3**

a)


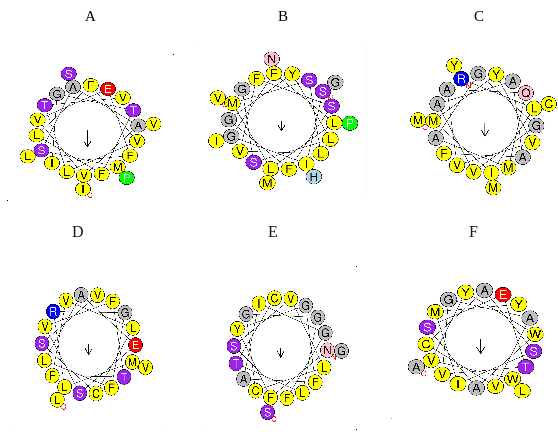


b)


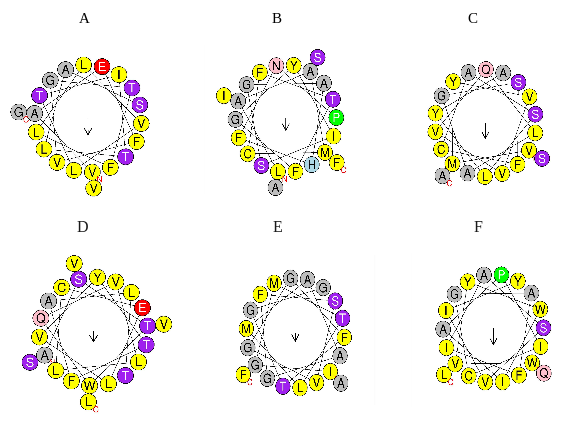


c)


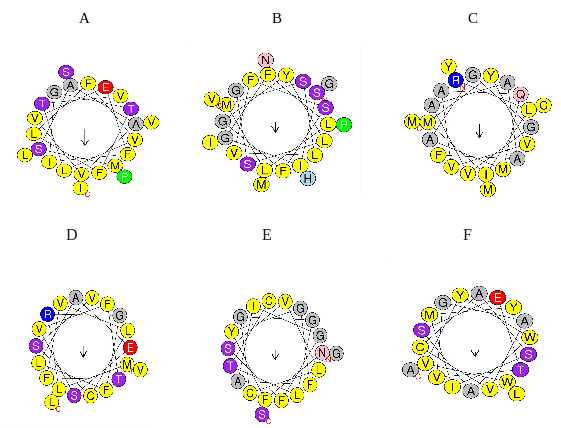
d)


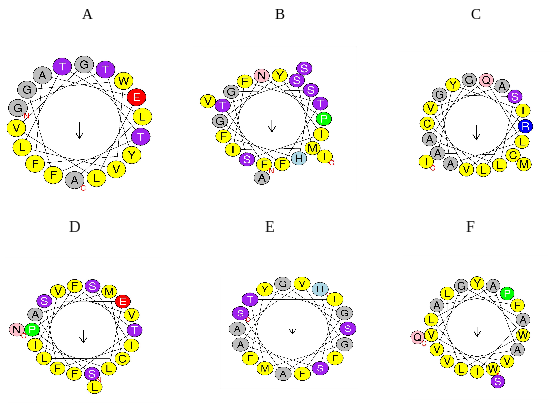


e)


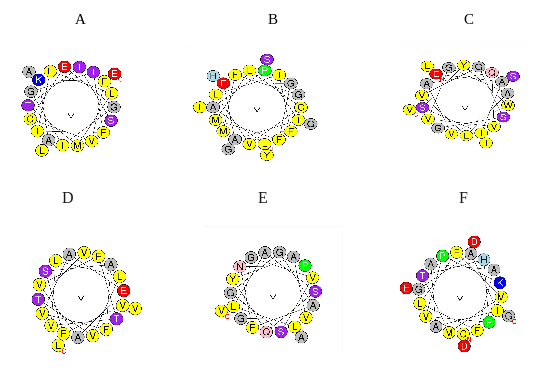

Supplement: Figure S3 — HeliQuest output for L. donovani . AQPs a) LdAQP1 b) LdAQP9, c) LdAQP putative d) LdAQP2860 e) LdAQP2870. Regions 1 to 6 have been shown in the form of helical wheel from A to F respectively. The best helical wheels, with medium hydrophobicity and high hydrophobic moment have been shown for each transmembrane region. Yellow region shows the hydrophobic face of helix. (DOCX) [file pone.0024820.s003.docx]

**Figure S4**

a.i.)


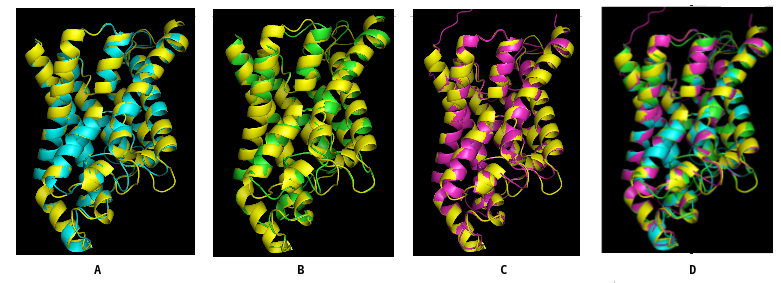


a.ii.)


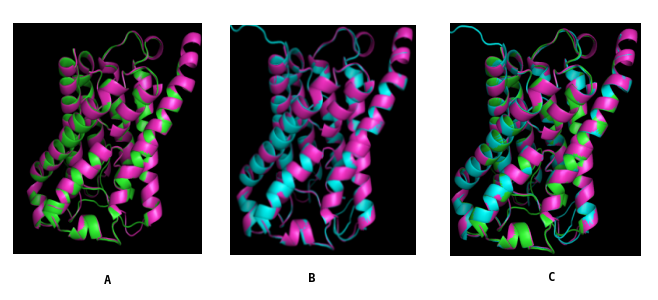
b)


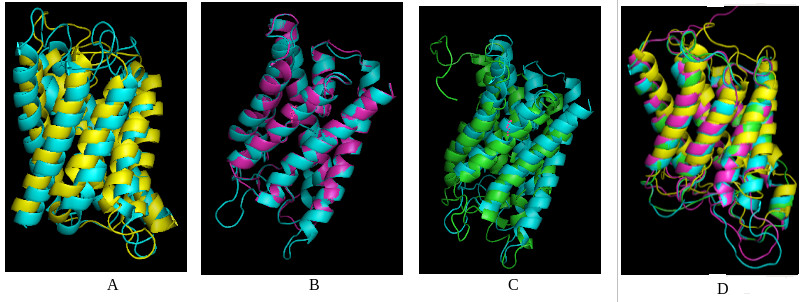
c)


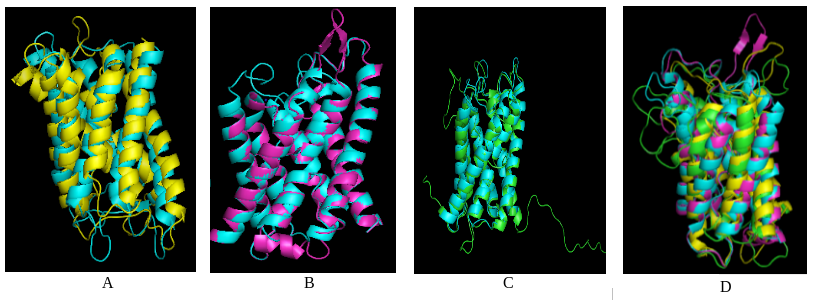
d.i)


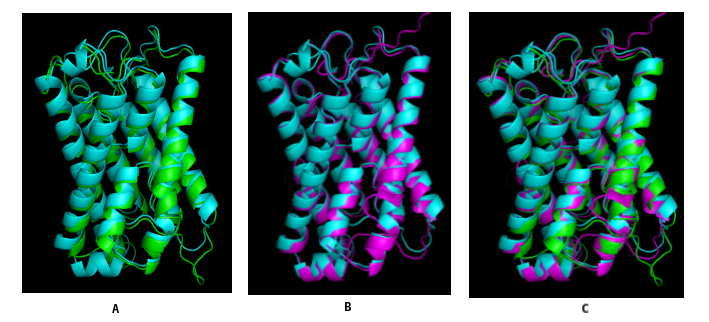
d.ii)


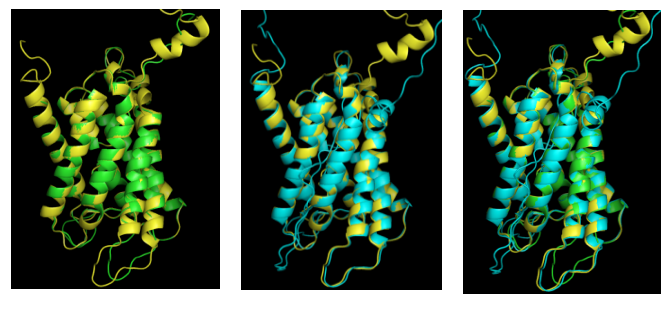
e)


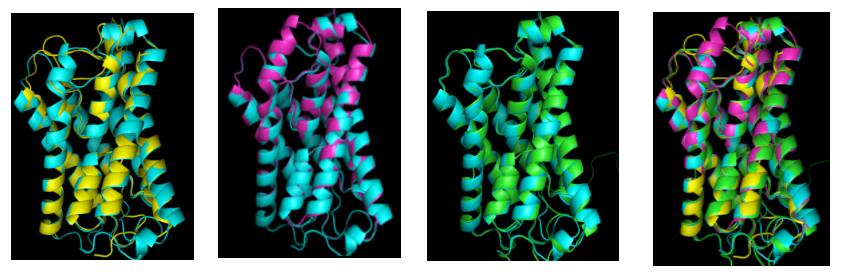

Supplement: Figure S4 — Structure predicted for L. donovani AQPs by A. EsyPred3D, B. 3D Jigsaw, C. MODELLER9v8. D. All three predicted models aligned with template. a) LdAQP1 a.i.) Template: E. coli AQGP [PDB ID: 1LDA, Resolution: 2.8 A°] a.ii.) Template: P. falciparum AQP* [PDB ID: 3C02, Resolution: 2.05°] b) LdAQP9 [Template: E.coli AQP, PDB ID: 2ABM, Resolution: 3.2 A°], c) LdAQP putative [Template: E. coli AQP, PDB ID: 2ABM, Resolution: 3.2 A°] d) LdAQP2860 d.i.) Template: Spinach AQP* [PDB ID: 1Z98, Resolution: 2.10 A°] and d.ii.) Template: Yeast AQP* [PDB ID: 2W2E, Resolution: 1.15 A°] e) LdAQP2870 [Template: Spinach AQP, PDB ID: 1Z98, Resolution: 2.10 A°]. *For these sequences the 3Djigsaw prediction was not possible due to poor sequence alignment. Hence the third image in a.ii), d.i), d.ii) are the alignment of both predicted models over the template. (DOCX) [file pone.0024820.s004.docx]

**Figure S5**


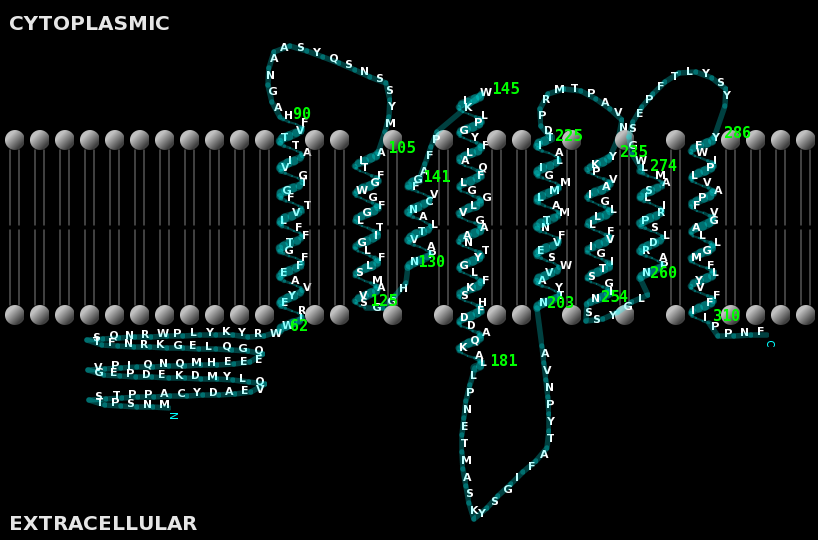
a)

b)


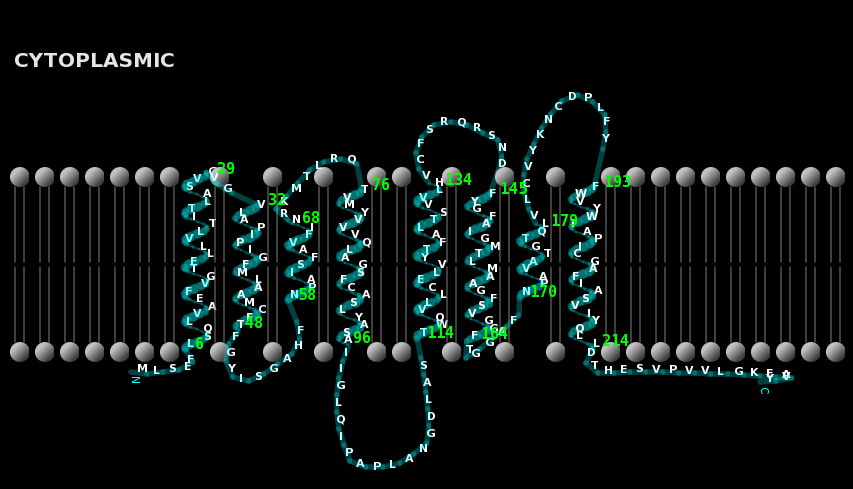


c)


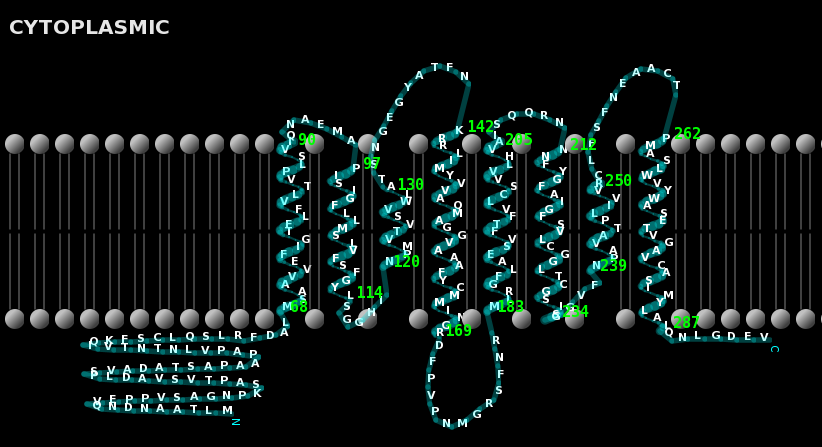


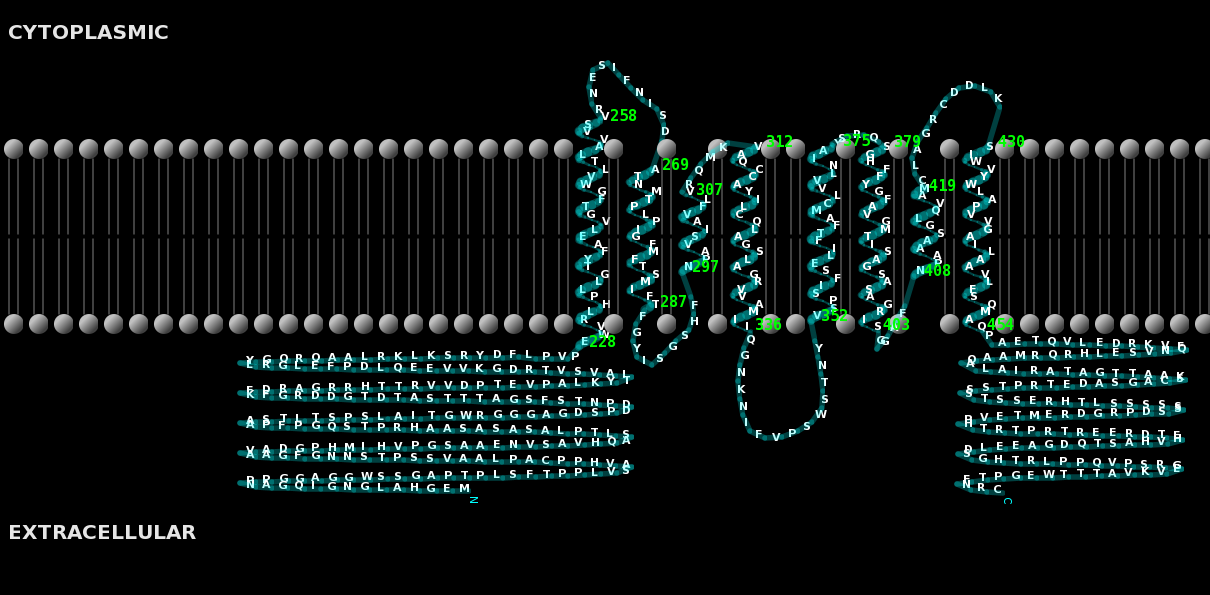
d)


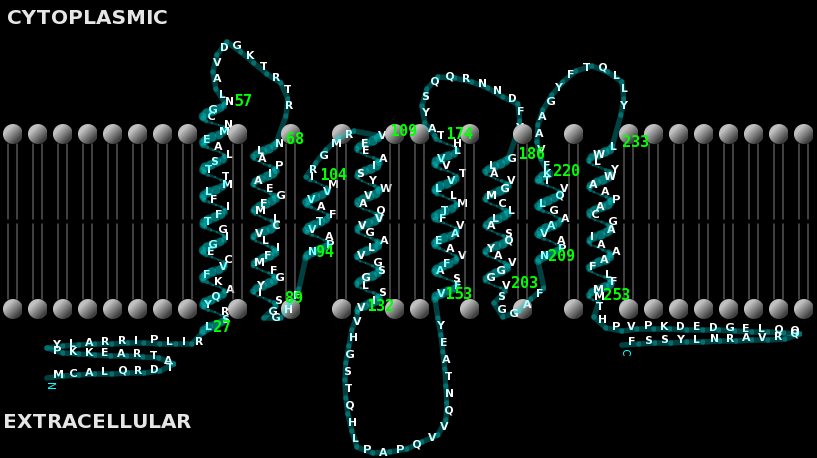
e)

Supplement: Figure S5 — The 2 D topology of predicted structure for Leishmania donovani AQPs. a) LdAQP1 b) LdAQP9, c) LdAQP putative d) LdAQP2860 e) LdAQP2870, showing the six major transmembrane helices and two small helices. (DOCX) [file pone.0024820.s005.docx]

**Figure S6**


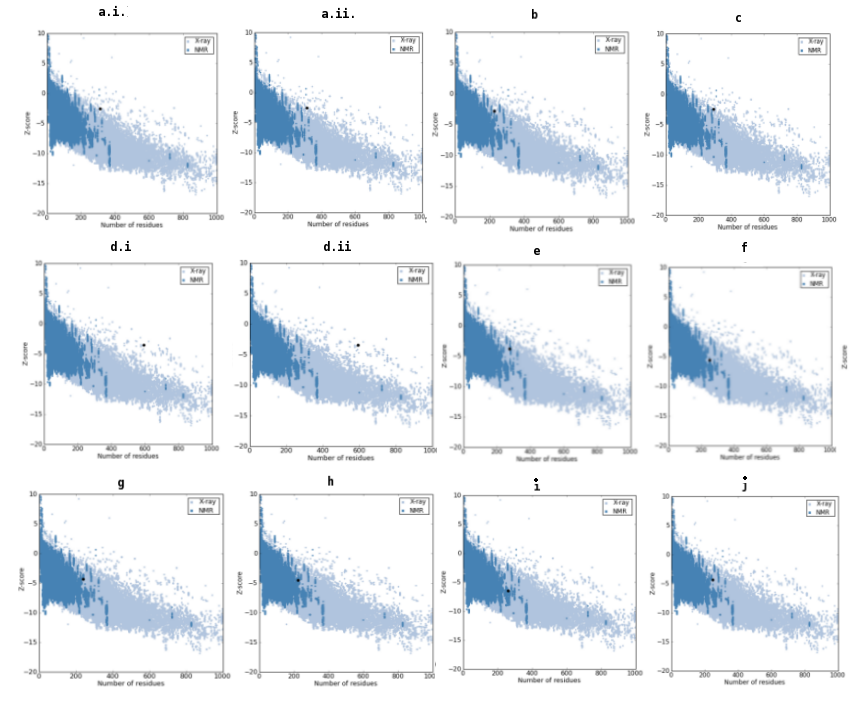

Supplement: Figure S6 — ProSAweb output for L. donovani AQPs predicted using MODELLER9v8 has been marked with a black dot. a) LdAQP1 a.i.) built using template E. coli AGP [Z-Score: −2.59] a.ii.) built using template P. falciparum AQP [Z-score: −2.60] b) LdAQP9 built using E. coli AQP [Z-Score: −2.84], c) LdAQP putative built using E. coli AQP [Z-Score: −2.53] d) LdAQP 2860 d.i) built using spinach AQP [Z-score: −3.61] d.ii.) built using yeast AQP [Z-Score: −3.45] e) LdAQP 2870 built using spinach AQP [Z-Score: −3.81]. In addition to the predicted models, the Z scores obtained for the templates are also shown. f) E. coli AQGP [PDB ID: 1LDA,Z-Score: −5.61] g) P. falciparum AQP [PDB ID: 3C02, Z-Score: −4.37] h) E. coli AQP [PDB ID: 2ABM, Z-Score: −4.55] i) Yeast AQP [PDB ID: 2W2E, Z-Score: −6.54] j) Spinach AQP [PDB ID: 1Z98, Z-Score: −4.36]. ProSAweb z-scores of all protein chains in PDB determined by X-ray crystallography are shown in light blue, whereas, those derived using NMR spectroscopy are shown in dark blue. (DOCX) [file pone.0024820.s006.docx]

**Figure S7**

a.i.)


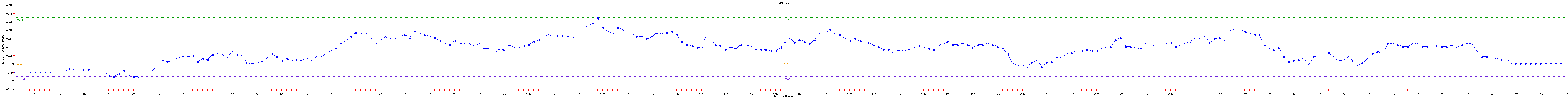
a.ii.)


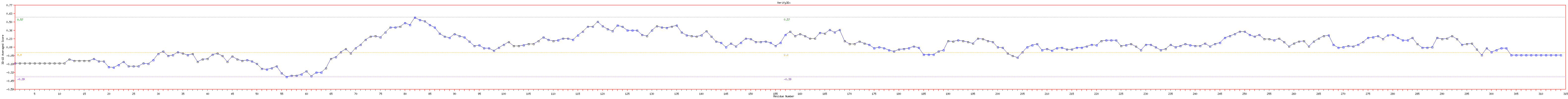
b)


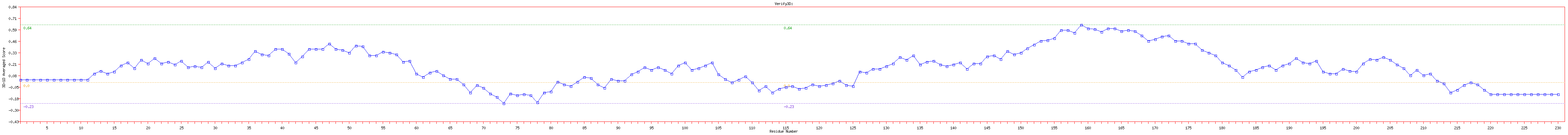
c)


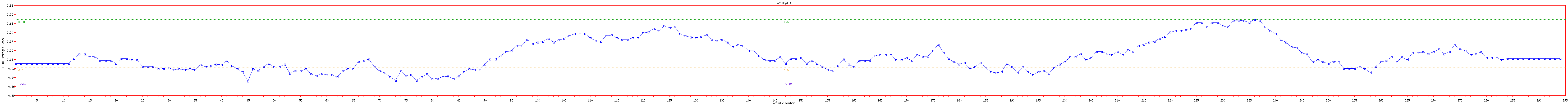


d.i.)


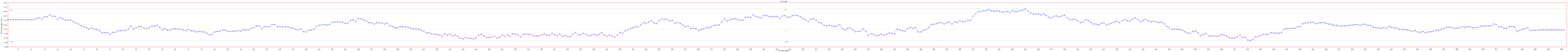


d.ii.)


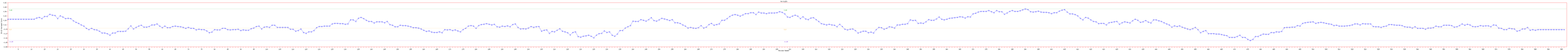


e)

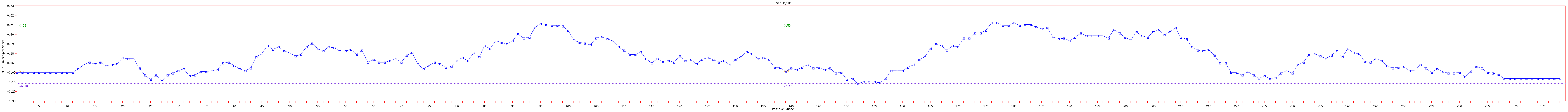


f)


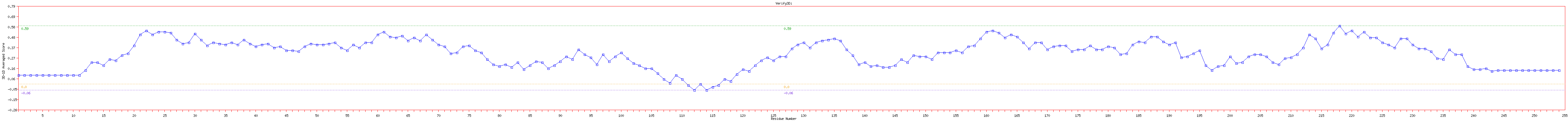


g)


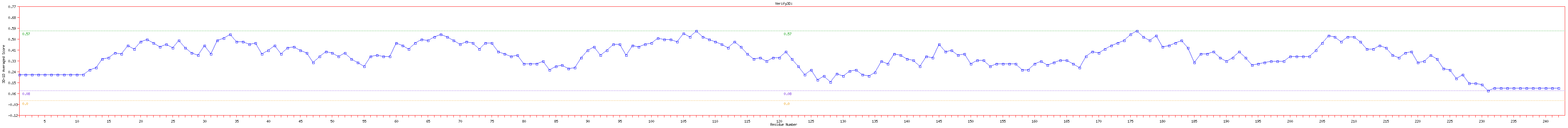


h)


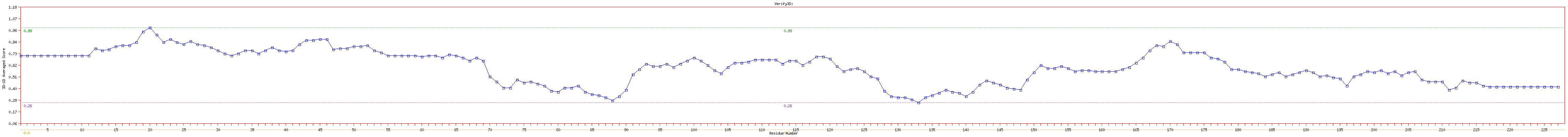


i)


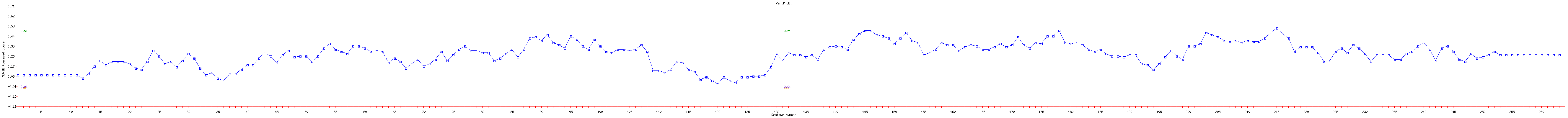


j)


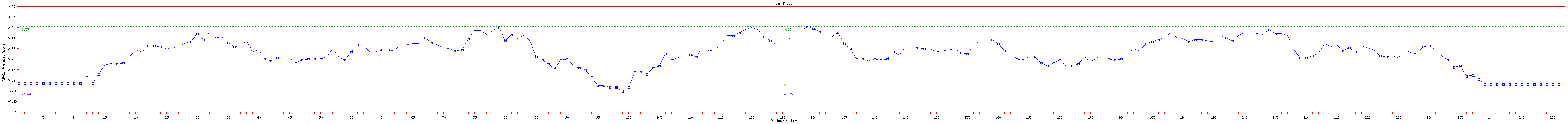

Supplement: Figure S7 — Verify3D output for L. donovani AQPs predicted using MODELLER9v8, a) LdAQP1 a.i.) built using template E. coli AGP a.ii.) built using template P. falciparum AQP b) LdAQP9 built using E. coli AQP c) LdAQP putative built using E. coli AQP d) LdAQP 2860 d.i) built using spinach AQP d.ii.) built using yeast AQP e) LdAQP 2870 built using spinach AQP. In addition to the predicted models, the plots obtained for the templates are also shown. f) E. coli AQGP [PDB ID: 1LDA] g) P. falciparum AQP [PDB ID: 3C02] h) E. coli AQP [PDB ID: 2ABM] i) Yeast AQP [PDB ID: 2W2E] j) Spinach AQP [PDB ID: 1Z98]. The score (accuracy in prediction) for the full sequence has been shown on Y axis, however, X axis has residue numbers. Green line marks the highest score, while orange line denotes the zero score, and purple shows lowest score in prediction. (DOCX) [file pone.0024820.s007.docx]

**Figure S8**

a.i.) a.ii.)


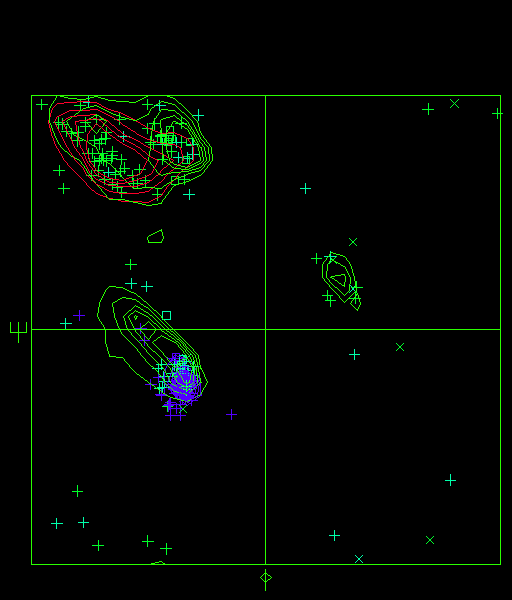

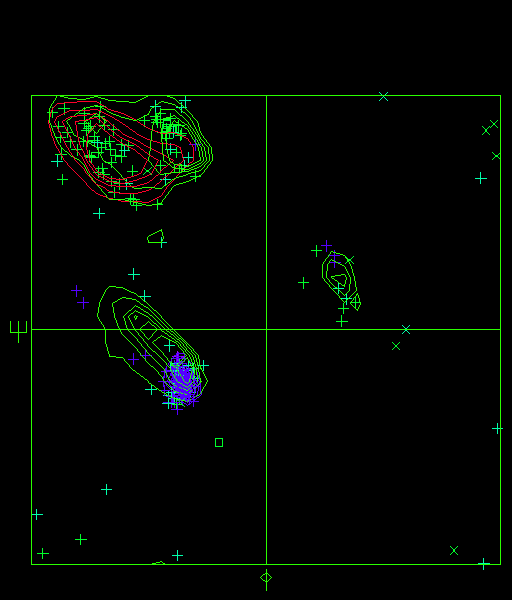


b) c)


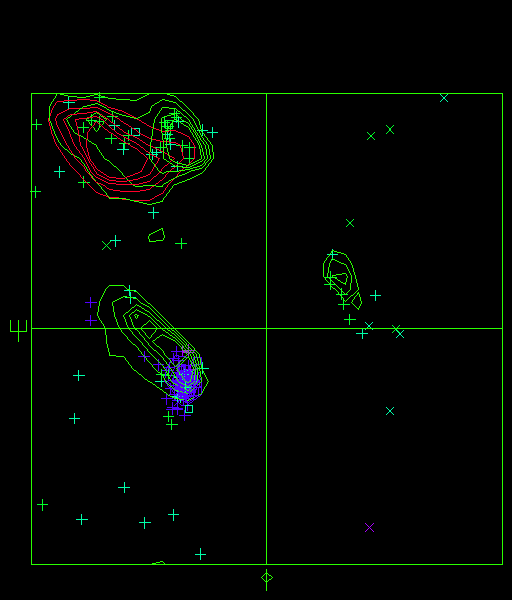

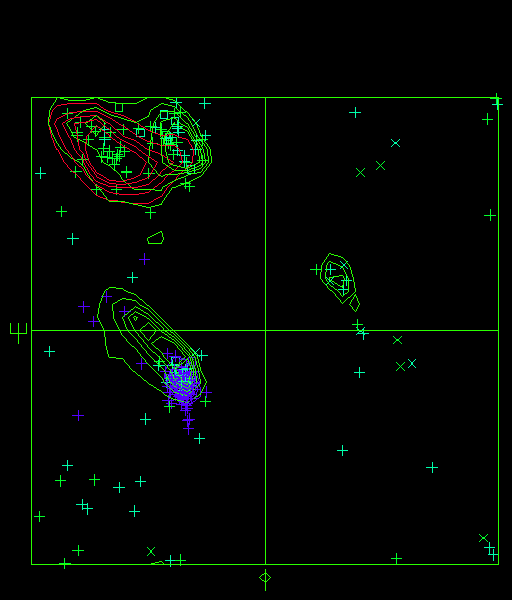


d.i.) d.ii.)


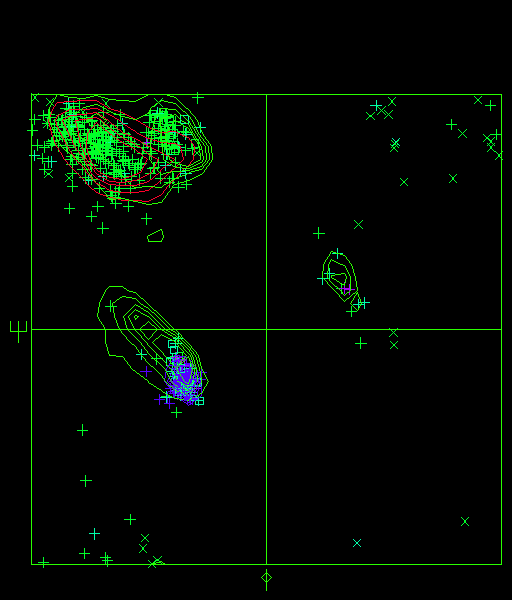

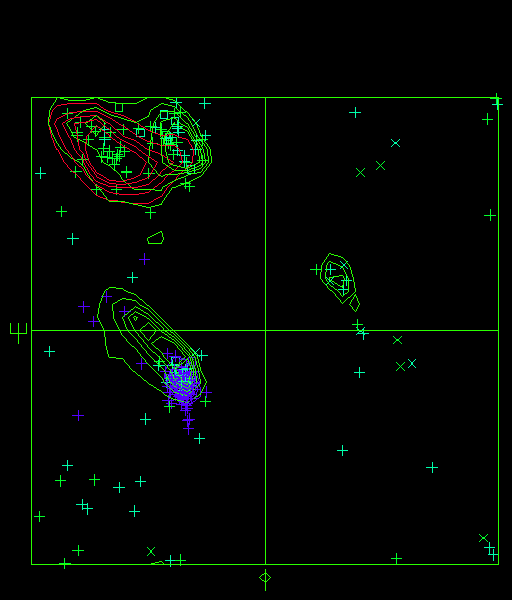


e) f)


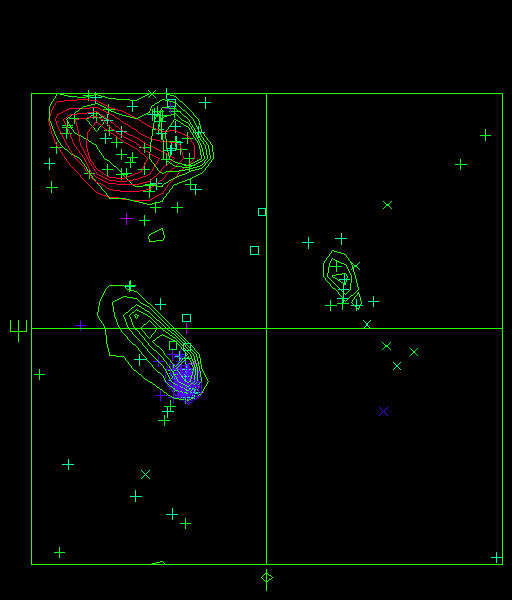

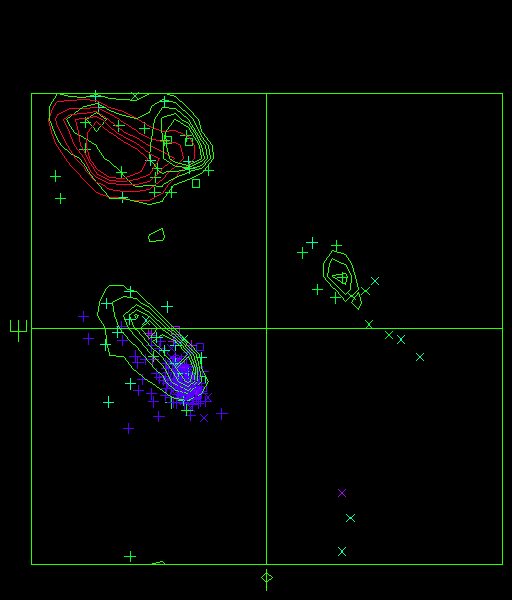


g) h)


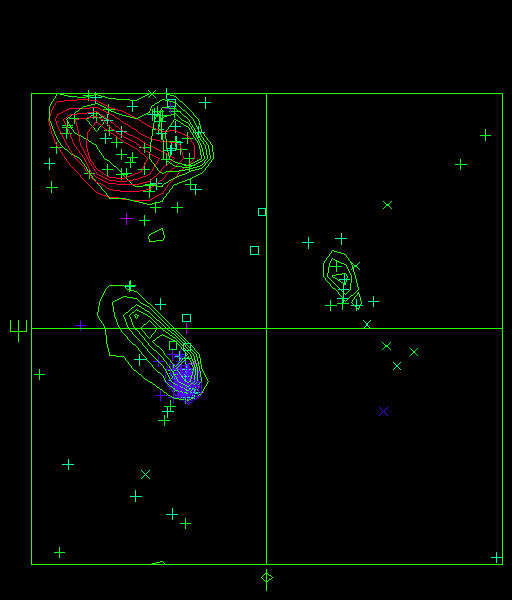

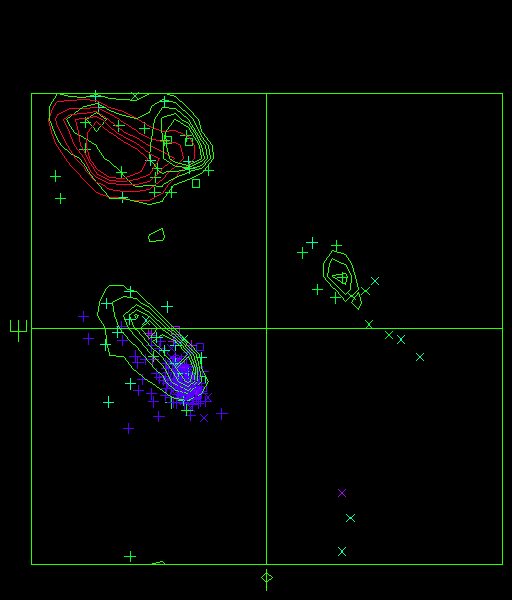


i) j)


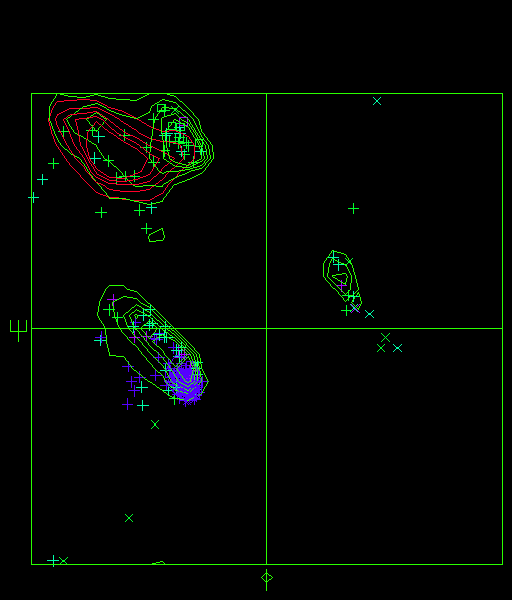

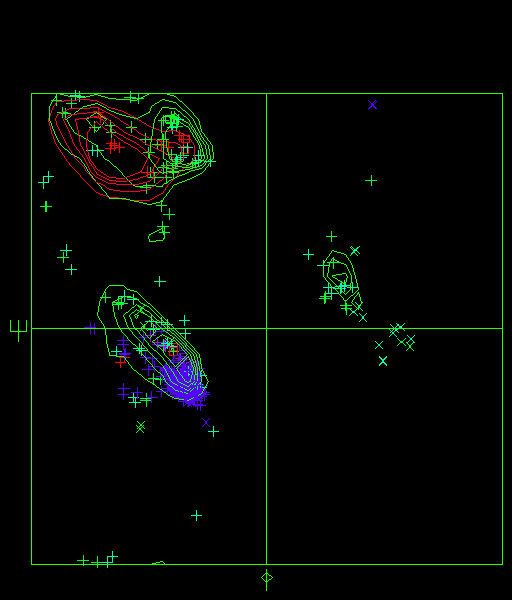

Supplement: Figure S8 — Validation of the MODELLER9v8 predicted structure using WHAT IF for L. donovani AQPs, a) LdAQP1 a.i.) built using template E. coli AGP a.ii.) built using template P. falciparum AQP b) LdAQP9 built using E. coli AQP c) LdAQP putative built using E. coli AQP d) LdAQP2860 d.i) built using spinach AQP d.ii.) built using yeast AQP e) LdAQP2870 built using spinach AQP. In addition to the predicted models, the plots obtained for the templates are also shown. f) E. coli AQGP [PDB ID: 1LDA] g) P. falciparum AQP [PDB ID: 3C02] h) E. coli AQP [PDB ID: 2ABM] i) Yeast AQP [PDB ID: 2W2E] j) Spinach AQP [PDB ID: 1Z98]. Here, blue means helix, red means strand and green means turn and loop (according to DSSP). The lines in the plot indicate prefered areas. The outer lines encircle the area within which 90% of all crosses of the same colour should be found; the inner lines indicate the 50% area. Orthogonal crosses indicate ‘normal’ residues; diagonal crosses indicate glycines and open squares indicate prolines. (DOCX) [file pone.0024820.s008.docx]

**Figure S9**

a.i)


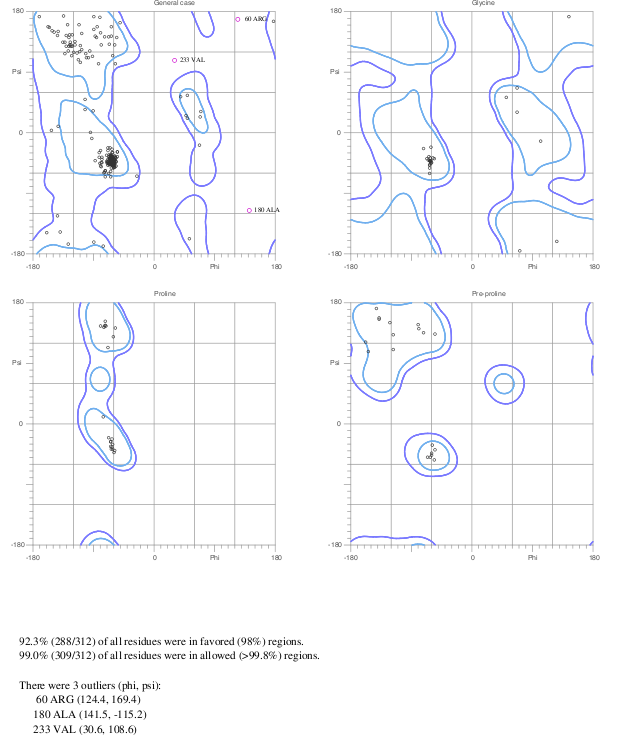


a.ii)


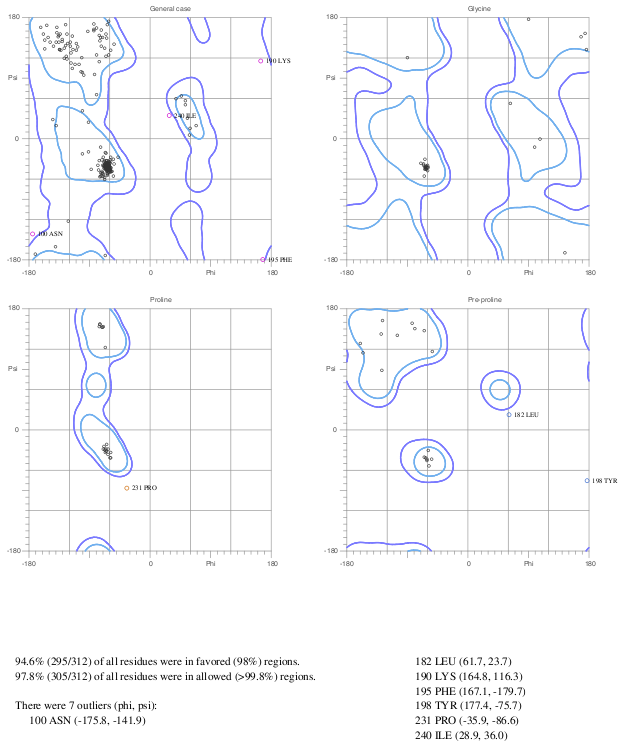


b)


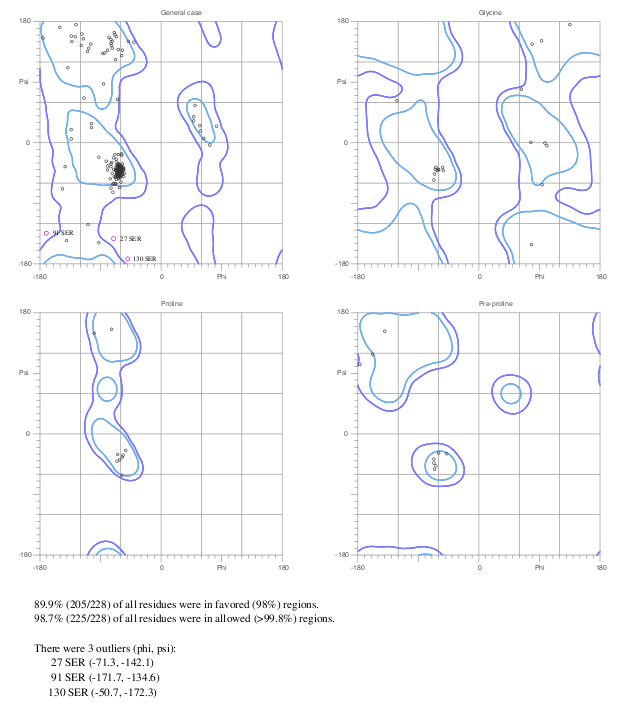


c)


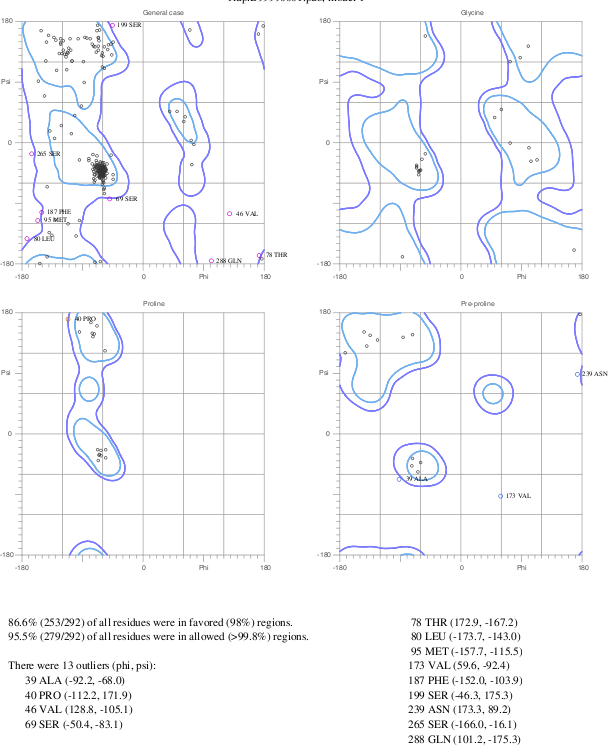


d.i.)


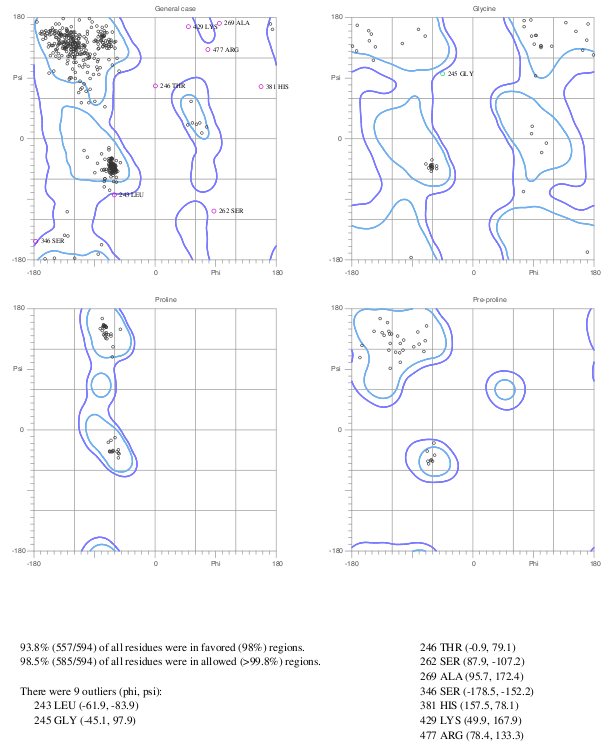


d.ii.)


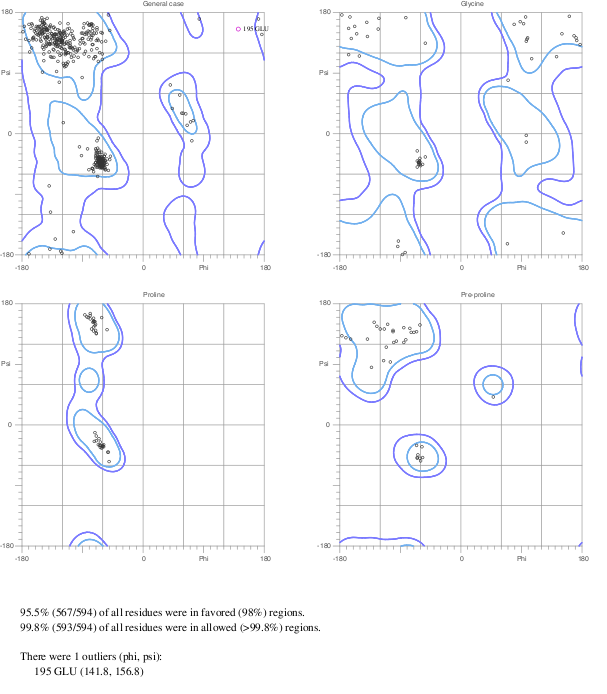


e)


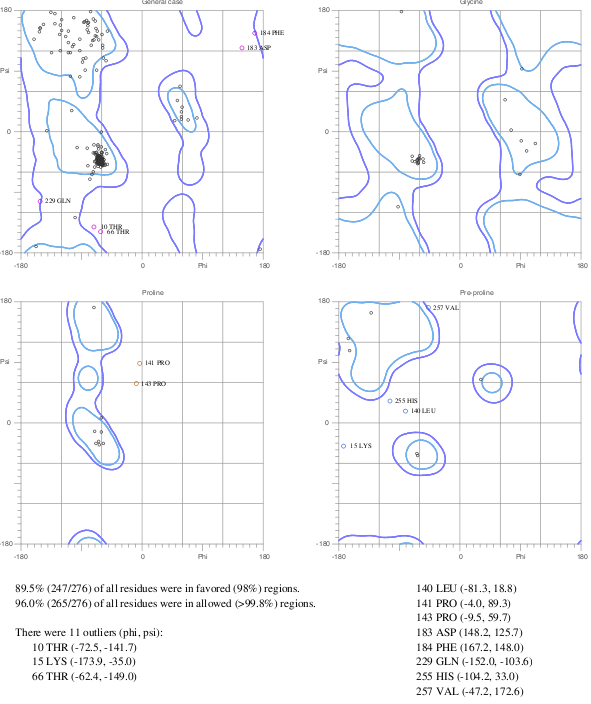


f)


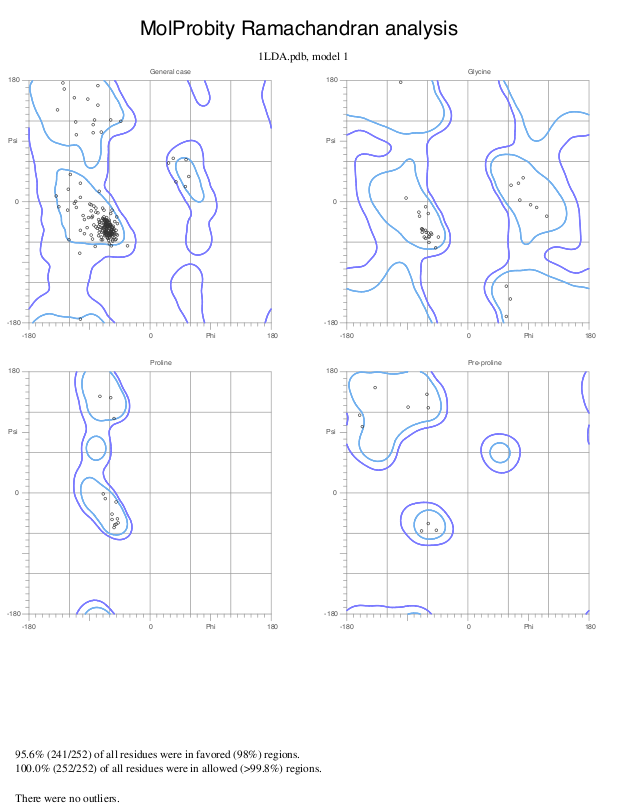


g)


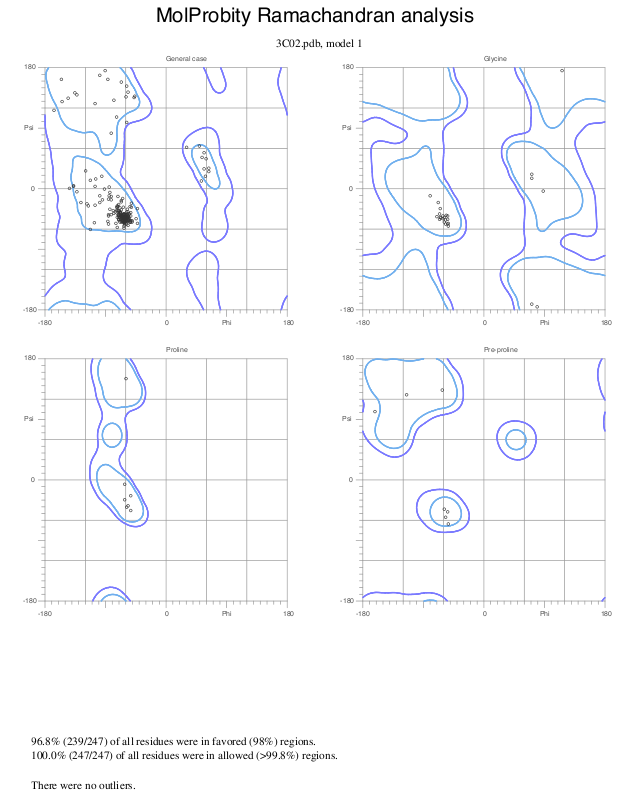


h)


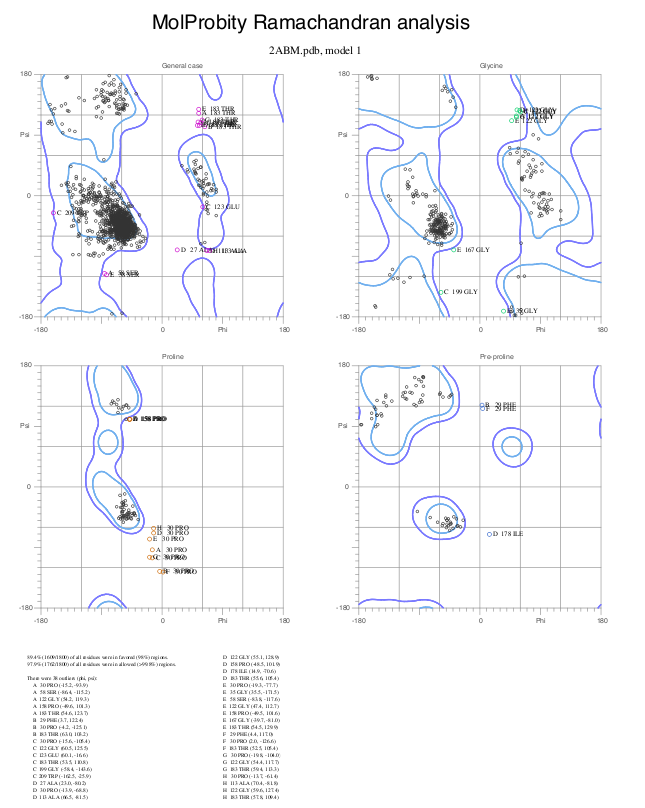


i)


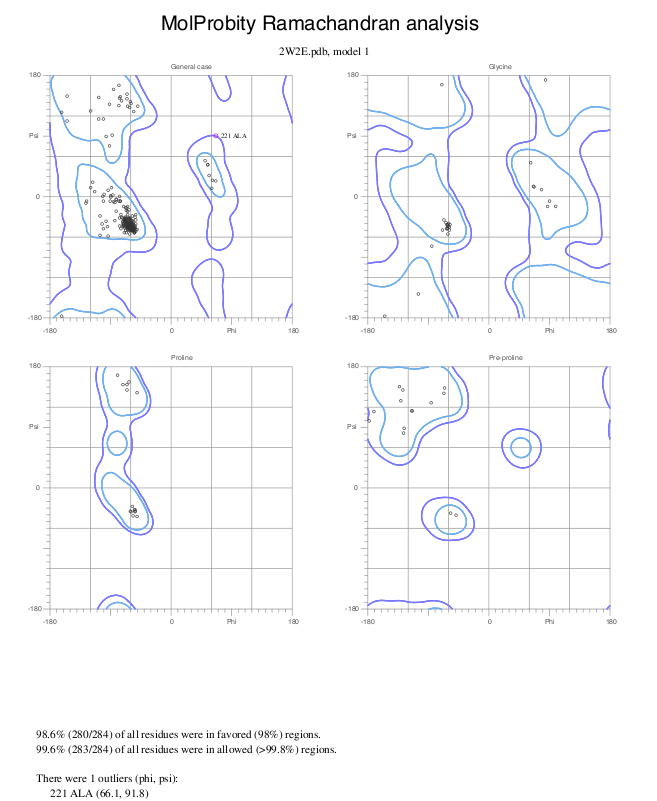


j)


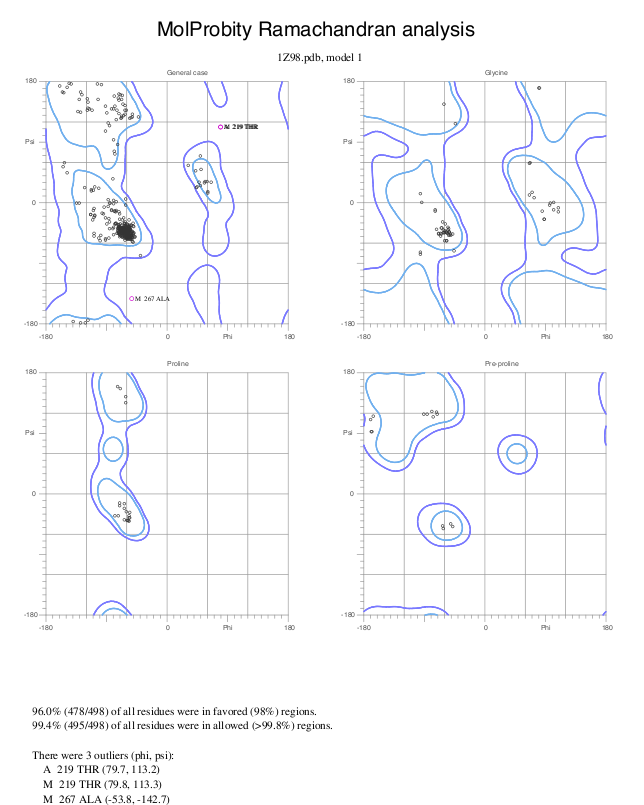

Supplement: Figure S9 — Validation of the MODELLER9v8 structures using MolProbity for L. donovani AQPs, a) LdAQP1 a.i.) built using template E. coli AGP a.ii.) built using template P. falciparum AQP b) LdAQP9 built using E. coli AQP c) LdAQP putative built using E. coli AQP d) LdAQP2860 d.i) built using spinach AQP d.ii.) built using yeast AQP e) LdAQP2870 built using spinach AQP. In addition to the predicted models, the plots obtained for the templates are also shown. f) E. coli AQGP [PDB ID: 1LDA] g) P. falciparum AQP [PDB ID: 3C02] h) E. coli AQP [PDB ID: 2ABM] i) Yeast AQP [PDB ID: 2W2E] j) Spinach AQP [PDB ID: 1Z98]. Ramachandran plot was obtained showing allowed/correct conformations in the predicted model. This analysis also gave the residue numbers that have incorrect conformations as outliers. (DOCX) [file pone.0024820.s009.docx]

**Figure S10**


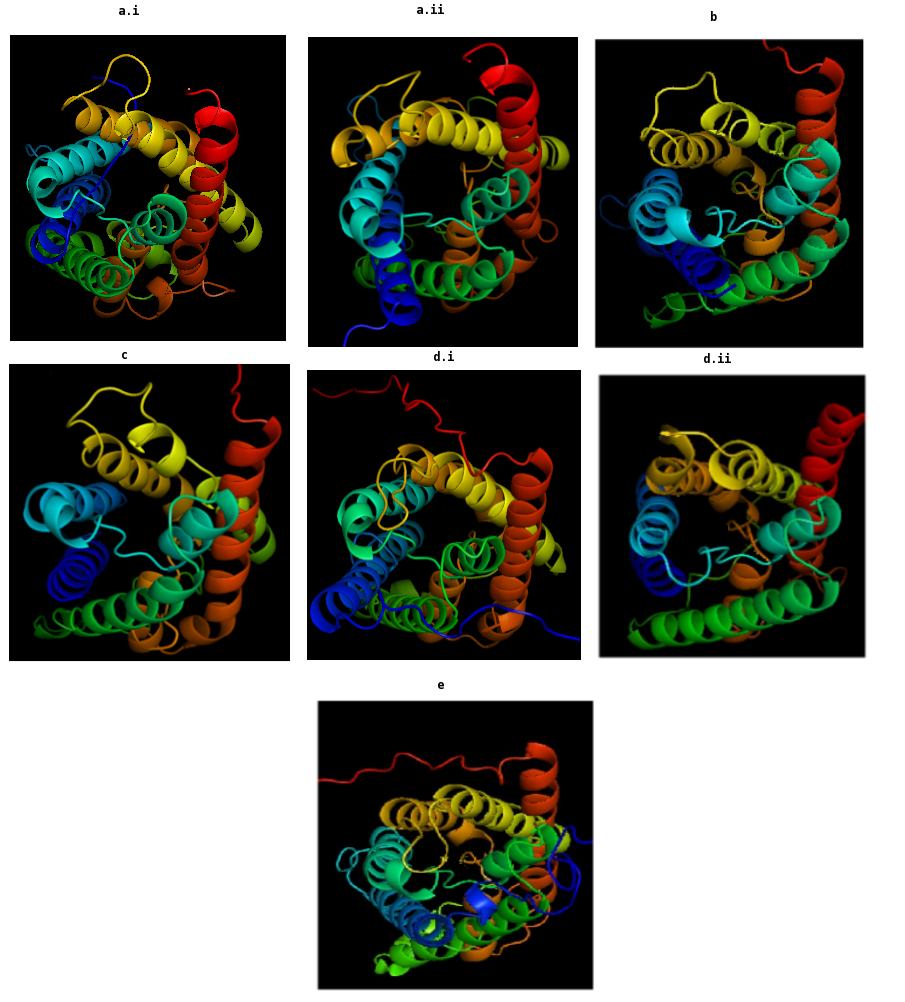

Supplement: Figure S10 — Top-down view of MODELLER9v8 structures for L. donovani AQPs, LdAQP1 a.i.) built using template E. coli AGP a.ii.) built using template P. falciparum AQP b) LdAQP9 built using E. coli AQP c) LdAQP putative built using E. coli AQP d) LdAQP2860 d.i) built using spinach AQP d.ii.) built using yeast AQP e) LdAQP2870 built using spinach AQP. The first major transmembrane helix in shown in dark blue, second in light blue, third in bright green, fourth in yellow, fifth in light orange and sixth in red. The two small helical regions hosting the NPA motif are shown in light green (present in between major helix 2 and 3) and orange (present in between major helix 5 and 6). (DOCX) [file pone.0024820.s010.docx]
