# Supplementary material for: Characterization of Leishmania donovani Aquaporins Shows Presence of Subcellular Aquaporins Similar to Tonoplast Intrinsic Proteins of Plants
Source: PLoS One. 2011 Sep 28;6(9):e24820. doi: 10.1371/journal.pone.0024820 (PMC3182166; doi:10.1371/journal.pone.0024820)
Supplement: Figure S11 — Porewalker predicted pore diameter at 3 A° in LdAQPs. a) LdAQP1 a.i.) built using template E. coli AQGP a.ii.) built using template P. falciparum AQP b) LdAQP9 built using E. coli AQP c) LdAQP putative built using E.coli AQP d) LdAQP2860 d.i) built using spinach AQP d.ii.) built using yeast AQP e) LdAQP2870 built using spinach AQP. In addition to the predicted models, the plots obtained for the templates are also shown. f) E. coli AQGP [PDB ID: 1LDA] g) P. falciparum AQP [PDB ID: 3C02] h) E. coli AQP [PDB ID: 2ABM] i) Yeast AQP [PDB ID: 2W2E] j) Spinach AQP [PDB ID: 1Z98]. PoreWalker is unable to handle too long queries, as in case of d.i), d.ii). (DOCX) [file pone.0024820.s011.docx]

**Figure S11**
