# Supplementary material for: Characterization of Leishmania donovani Aquaporins Shows Presence of Subcellular Aquaporins Similar to Tonoplast Intrinsic Proteins of Plants
Source: PLoS One. 2011 Sep 28;6(9):e24820. doi: 10.1371/journal.pone.0024820 (PMC3182166; doi:10.1371/journal.pone.0024820)
Supplement: Table S1 — AQPs from different species with reported crystal structures. (DOCX) [file pone.0024820.s014.docx]

Table S1

| **Specie** | **PDB ID** | **Resolution** | **Reference** |
| --- | --- | --- | --- |
| Human AQP1 | 1IH5 | 3.70 A° | Ren et al, 2001 |
| Human AQP4 | 3GD8 | 1.80 A° | Ho et al, 2009 |
| Human AQP5 | 3D9S | 2.00 A° | Horsefield et al, 2008 |
| E. coli AQP | 2ABM | 3.20 A° | Jiang et al, 2006 |
| E.coli AQGP (GlpF) | 1LDA | 2.80 A° | Tajkhorshid et al, 2002 |
| Yeast AQP | 2W2E | 1.15 A° | Fischer et al, 2009 |
| Spinach (SoPIP2) | 1Z98 | 2.10 A° | Horsefield et al, 2006 |
| Rat AQP4 | 2ZZ9 | 2.80 A° | Hiroaki et al, 2005 |
| P. falciparum | 3C02 | 2.05 A° | Newby et al, 2008 |
