# Supplementary material for: Characterization of Leishmania donovani Aquaporins Shows Presence of Subcellular Aquaporins Similar to Tonoplast Intrinsic Proteins of Plants
Source: PLoS One. 2011 Sep 28;6(9):e24820. doi: 10.1371/journal.pone.0024820 (PMC3182166; doi:10.1371/journal.pone.0024820)
Supplement: Table S2 — (a) RMSD values for models predicted using MODELLER9v8 for all L. donovani AQPs with structural templates: P. falciparum [PDB ID: 3C02, chain A, Resolution: 2.05 A°], E. coli AQGP [PDB ID: 1LDA, chain A, Resolution: 2.8 A°], E. coli AQP [PDB ID: 2ABM, chain A, Resolution: 3.2 A°], Spinach AQP [PDB ID: 1Z98, chain A, Resolution: 2.10 A°], Yeast AQP [PDB ID: 2W2E, chain A, Resolution: 1.15 A°]. b: RMSD values for all models predicted using EsyPred3D, 3Djigsaw and MODELLER9v8 for five L. donovani AQPs with various structural templates: P. falciparum [PDB ID: 3C02, chain A, Resolution: 2.05 A°], E. coli AQGP [PDB ID: 1LDA, chain A, Resolution: 2.8 A°], E. coli AQP [PDB ID: 2ABM, chain A, Resolution: 3.2 A°], Spinach AQP [PDB ID: 1Z98, chain A, Resolution: 2.10 A°], Yeast AQP [PDB ID: 2W2E, chain A, Resolution: 1.15 A°]. (DOCX) [file pone.0024820.s015.docx]

Table S2 a

| **Query** | **RMSD values of predicted model from the structural template** | | | | |
| --- | --- | --- | --- | --- | --- |
| ***L.donovani* AQP** | **P.fal AQP** | **Ecoli AQGP** | **Ecoli AQP** | **Spinach AQP** | **Yeast AQP** |
| LdAQP 1 | 0.176 | 0.361 | 2.411 | - | - |
| LdAQP 9 | - | - | 0.566 | 1.437 | - |
| LdAQP putative | - | - | 1.791 | 2.625 | - |
| LdAQP 2860 | - | - | 4.016 | 1.865 | 0.756 |
| LdAQP 2870 | - | - | 17.866 | 0.712 | - |

**The trend in RMSD values shows that apart from the sequence similarity shown in the phylogram, the resolution of the structural template plays an important role in building of a homology model. Hence, P.fal AQP is a better structural template than Ecoli AQGP for building LdAQP1. Also, Yeast AQP which has a higher crystal structure resolution, serves as a better structural template for LdAQP2860 than Ecoli AQP or spinach AQP.


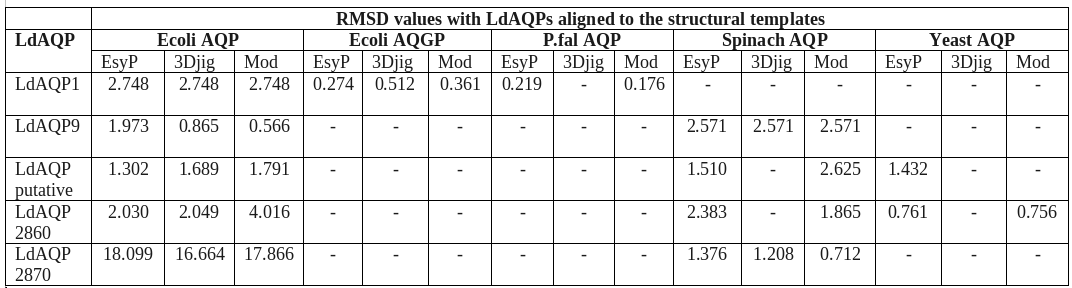
Table S2 b
