# Supplementary material for: Characterization of Leishmania donovani Aquaporins Shows Presence of Subcellular Aquaporins Similar to Tonoplast Intrinsic Proteins of Plants
Source: PLoS One. 2011 Sep 28;6(9):e24820. doi: 10.1371/journal.pone.0024820 (PMC3182166; doi:10.1371/journal.pone.0024820)
Supplement: Table S3 — (A) List of Primers used (restriction enzyme sites underlined) for getting PCR products that were cloned into pESC-URA (yeast expression vector). b: List of Primers used (restriction enzyme sites underlined) for getting PCR products that were cloned into Leishmania specific-GFP vectors c: List of Primers used for Real time PCR. (DOCX) [file pone.0024820.s016.docx]

Table S3 a

| **Oligo name** | **Primer sequence (5’-3’)** | **Amplicon size (bp) and Annealing temperature** |
| --- | --- | --- |
| AQP9 F | CGGGATCCTATGCTCTCCGAGTTCCTCAGT | 693, 57 °C |
| AQP9 R | ACGCGTCGACATACACCGCTTCCTTGCCTA |  |
| AQP Put. F | CGGGATCCTATGCTTACTGCCGCGAAC | 885, 53 °C |
| AQP Put. R | ACGCGTCGACGACCTCATCCCCCAAGTTC |  |
| AQP 2870 F | CGGGATCCTATGTGCGCTCTGCAGAGG | 837, 57 °C |
| AQP 2870 R | ACGCGTCGACGAAAGAGCTGTATAGATTGCGTG |  |
| AQP1 F | GAAGATCTTATGAACTCTCCTACAAGCACA | 945, 60 °C |
| AQP1 R | ACGCGTCGACGAAGTTGGGTGGAATGA |  |

Table S3 b

| **Oligo name** | **Primer sequence (5’-3’)** | **Amplicon size (bp) and annealing temperature** | **Vector** |
| --- | --- | --- | --- |
| AQP9 F | CCCAAGCTTGGGATGCTCTCCGAGTTCCTCAGT | 690, 57ºC | pGEM-7zfαNeoαGFP |
| AQP9 R | CCCAAGCTTGGGATACACCGCTTCCTTGCCTA |  |  |
| AQP Put. F | CGGGATCCTATGCTTACTGCCGCGAAC | 882, 53ºC | pSP-72αneoαGFP |
| AQP Put. R | GCTCTAGAGCGACCTCATCCCCCAAGTTC |  |  |
| AQP2870 F | CGGGATCCATGTGCGCTCTGCAGAGG | 834, 57ºC | pSP-72αneoαGFP |
| AQP2870 R | GCTCTAGAGAAAGAGCTGTATAGATTGCGTG |  |  |
| AQP1 F | CCCAAGCTTATGAACTCTCCTACAAGCACAC | 942, 60ºC | pGEM-7zfαNeoαGFP |
| AQP1 R | CCCAAGCTTGAAGTTGGGTGGAATGATGA |  |  |

**Table S3 c**

******
